# Diversity of Free-Living Environmental Bacteria and Their Interactions With a Bactivorous Amoeba

**DOI:** 10.3389/fcimb.2018.00411

**Published:** 2018-11-23

**Authors:** Debra A. Brock, Tamara S. Haselkorn, Justine R. Garcia, Usman Bashir, Tracy E. Douglas, Jesse Galloway, Fisher Brodie, David C. Queller, Joan E. Strassmann

**Affiliations:** ^1^Queller/Strassmann Laboratory, Washington University in St. Louis, Department of Biology, St. Louis, MO, United States; ^2^Mountain Lake Biological Laboratory, University of Virginia, Mountain Lake, VA, United States

**Keywords:** *Dictyostelium*, bacteria, microbiome, symbiosis, amoebae, sociality, persistence, predation

## Abstract

A small subset of bacteria in soil interact directly with eukaryotes. Which ones do so can reveal what is important to a eukaryote and how eukaryote defenses might be breached. Soil amoebae are simple eukaryotic organisms and as such could be particularly good for understanding how eukaryote microbiomes originate and are maintained. One such amoeba, *Dictyostelium discoideum*, has both permanent and temporary associations with bacteria. Here we focus on culturable bacterial associates in order to interrogate their relationship with *D. discoideum*. To do this, we isolated over 250 *D. discoideum* fruiting body samples from soil and deer feces at Mountain Lake Biological Station. In one-third of the wild *D. discoideum* we tested, one to six bacterial species were found per fruiting body sorus (spore mass) for a total of 174 bacterial isolates. The remaining two-thirds of *D. discoideum* fruiting body samples did not contain culturable bacteria, as is thought to be the norm. A majority (71.4%) of the unique bacterial haplotypes are in Proteobacteria. The rest are in either Actinobacteria, Bacteriodetes, or Firmicutes. The highest bacterial diversity was found in *D. discoideum* fruiting bodies originating from deer feces (27 OTUs), greater than either of those originating in shallow (11 OTUs) or in deep soil (4 OTUs). Rarefaction curves and the Chao1 estimator for species richness indicated the diversity in any substrate was not fully sampled, but for soil it came close. A majority of the *D. discoideum*-associated bacteria were edible by *D. discoideum* and supported its growth (75.2% for feces and 81.8% for soil habitats). However, we found several bacteria genera were able to evade phagocytosis and persist in *D. discoideum* cells through one or more social cycles. This study focuses not on the entire *D. discoideum* microbiome, but on the culturable subset of bacteria that have important eukaryote interactions as prey, symbionts, or pathogens. These eukaryote and bacteria interactions may provide fertile ground for investigations of bacteria using amoebas to gain an initial foothold in eukaryotes and of the origins of symbiosis and simple microbiomes.

## Introduction

Eukaryotes evolved in the context of a world already fully populated by bacteria and archaea (McFall-Ngai et al., 2013). Understanding the consequences and extent of this fairly recent realization is an enterprise still in its infancy. There are several different major approaches that include studies of the importance of gut microbiomes for food acquisition, plant root microbiomes for nutrient up-take, and the obligate symbioses of sap feeding insects and their bacteria (Moran et al., 2005; Alcock et al., 2014; Lareen et al., 2016). These studies and others are changing the way we view the place of eukaryotes in the living landscape. Important and paradigm changing as these studies are, they do not let us go back over two billion years to when eukaryotes were first adapting to the microbial world. Indeed, such a study would not be possible. However, we can make a closer approach to this goal by studying extant lineages from lineages more basal than animals and plants, though of course even their associations with bacteria still reflect modern adaptations and patterns.

Here, we use *Dictyostelium discoideum*, a single-celled bactivorous soil amoeba from a basal eukaryotic lineage, to investigate the range of interactions a micro-eukaryote has with bacteria in nature. The soil context is important because it is an environment where different organisms are in close contact with an abundance of potential bacterial prey. As such, our study organism exhibits a wide range of interactions with bacteria. First discovered as a predator of soil bacteria (Raper, 1937), *D. discoideum* has been employed as a model in many host-pathogen studies (Solomon et al., 2000; Alibaud et al., 2008; Hagedorn et al., 2009; Jia et al., 2009; Hasselbring et al., 2011), and has recently been found to engage in symbiotic associations with bacteria throughout their life history (Brock et al., 2011; DiSalvo et al., 2015). However, little is known about what bacteria *D. discoideum* associate with or prey upon in nature. The natural habitat of wild *D. discoideum* is deciduous forest soil and leaf litter and it is most commonly isolated from the acidic soils of eastern North America Appalachian forests (Cavender and Raper, 1965a,b; Raper, 1984; Landolt and Stephenson, 1986). Historically, Dictyostelids were first isolated from the dung of various herbivores (Raper, 1984) most notably from deer feces in North American forests (Stephenson and Landolt, 1992; Gilbert et al., 2007). Early work by Kenneth Raper indicated NC-4, the type-clone of *D. discoideum* isolated in the forests of North Carolina, could feed and develop on both gram-negative and gram positive non-pathogenic bacteria (Raper, 1937). In a later report, Raper and Smith also tested the growth of NC-4 against pathogenic bacteria species that may similarly occur in the soil, ascertaining that *D. discoideum* has a wide range of prey bacteria (Raper and Smith, 1939). More recently it has become clear that *D. discoideum* has different mechanisms for dealing with different prey species (Nasser et al., 2013).

*D. discoideum* can be thought of as a primitive macrophage capable of ingesting, killing and digesting at least one bacterium per minute as long as prey bacteria are available (Cosson and Soldati, 2008). Yet, upon depletion of available food, single-celled starving amoebae aggregate by the thousands using cAMP signals to form a multicellular organism (Kessin, 2001). During this transformation into a multicellular organism, *D. discoideum* slugs have some cells capable of immune-like functions (Chen et al., 2007; Brock et al., 2016a). These cells known as sentinel cells can remove bacterial pathogens as well as detoxify the slug to protect the presumptive spore population from harm. Slugs are motile and can migrate toward more favorable locations using light and heat as cues before terminally differentiating into a fruiting body. The fruiting body is composed a spherical sorus containing spores held aloft by a stalk to allow for spore dispersal. For decades, fruiting bodies containing the next generation of *Dictyostelium* spores were thought to be free from bacteria (Raper, 1937). However, some lineages of wild amoebae that we call farmers have stable interactions with different bacterial partners that are both food and weapons (Brock et al., 2011; Stallforth et al., 2013). Farmers have reduced sentinel cell numbers compared to *D. discoideum* clones without bacteria, suggesting a relaxation of innate immunity to allow carriage of bacterial associates (Brock et al., 2016a). We have reported two major clades of inedible *Burkholderia* as the most prominent bacterial partners of *D. discoideum* farmers (DiSalvo et al., 2015). These stable *Burkholderia* partners can colonize naïve *Dictyostelium* hosts and are the drivers of the proto-farming phenotype by allowing co-carriage of food bacteria through multiple social cycles.

To more completely explore the full nature and extent of the associations of a micro-eukaryote with bacteria in natural environments, we conducted a large-scale survey of soil and deer feces at Mountain Lake Biological Station in Virginia. We isolated wild *D. discoideum* to determine what, if any, culturable bacteria remain associated after *D. discoideum* amoebae differentiate into fruiting bodies. We focused on culturable bacteria in this survey to perform edibility and persistence assays to interrogate the type of interaction each bacterium had with wild *D. discoideum*. We found many different bacteria genera, which group into 32-species level OTUs (Operational Taxonomic Unit), transiently associated with the wild *D. discoideum* recovered from this survey. Here we detail the diversity of bacteria acquired by and maintained in *D. discoideum* throughout the social stage, and how bacterial diversity differs among different *D. discoideum* habitats. We show what these bacteria mean to their eukaryote host in the most ancient of relationships: predation and resistance of predation. The data presented here highlight the rich opportunities available to gain greater understanding of eukaryote-bacteria associations using a simple single-celled model organism.

## Materials and methods

### Deer feces and soil collection

**Figure 1:**
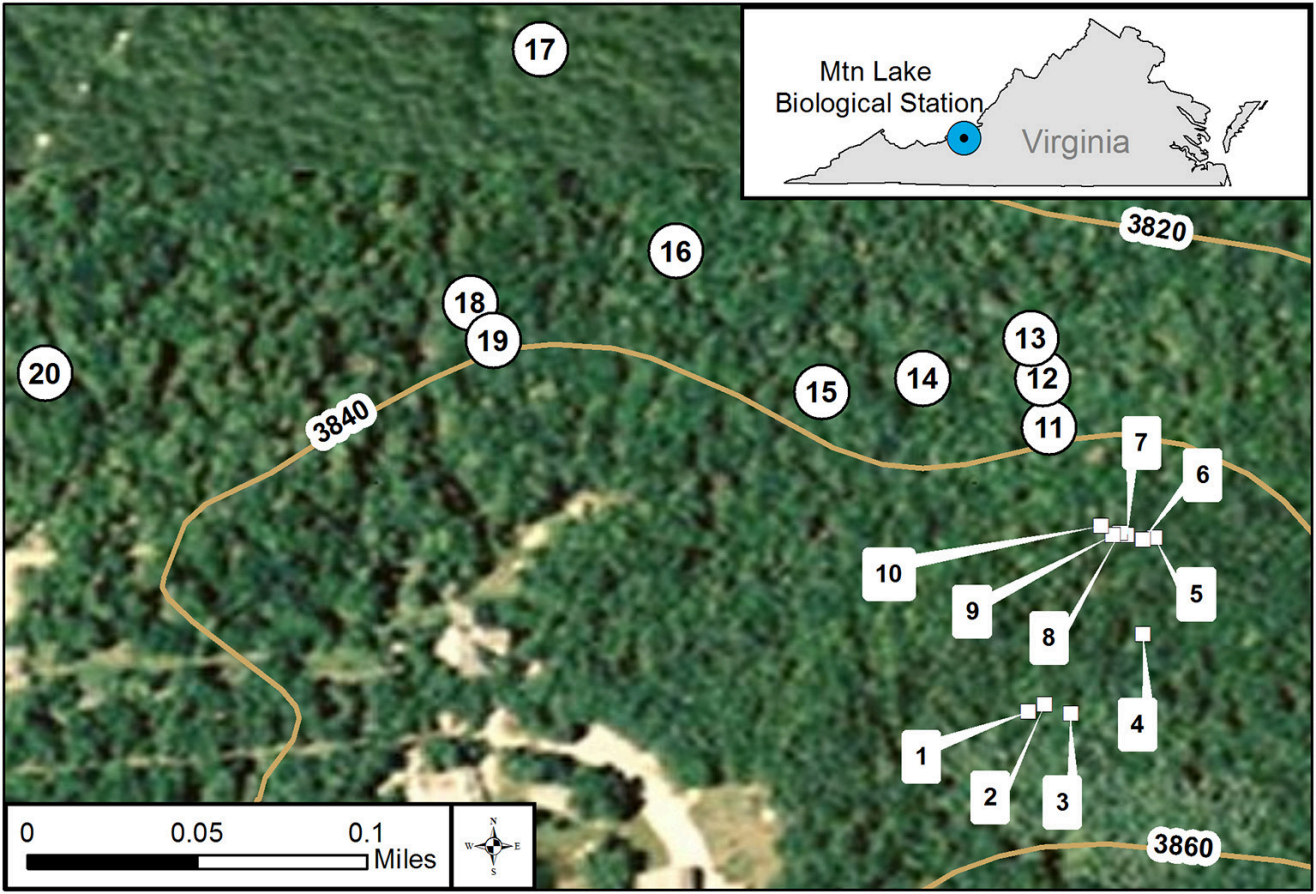
GIS map of individual collection locations at Mt. Lake Biological Station, VA. We used GIS (geographical information system) to create a map of the collection locations. We did collections on two dates in July 2014 represented by square symbols numbered 1–10 and the round symbols numbered 11–20. The curved lines represent various elevations at our site. The small inset map details the location of Mt. Lake Biological Station in the state of Virginia, USA.

We collected samples of feces from white-tailed deer (*Odocoileus virginianus*) and of soil on 23 and 30 July 2014 from mixed deciduous forests at Mountain Lake Biological Station, Virginia (37.375654° −80.522140°) at Salt Pond Mountain on the eastern continental divide in the Appalachian Mountains (see Figure 1 for collection locations and Supplementary Table 1 for GPS coordinates and dates). On each collection date, we first identified and collected ten separate piles of fresh deer feces. In addition to the feces, we collected two soil samples about 5 meters from each feces pile. One soil sample was shallow, just under the leaf litter about one to five centimeters deep. We collected the second soil sample about fifteen centimeters deep, adjacent to the shallow soil sample. We sterilized collection implements using ethanol wipes before and after each use to avoid cross contamination between sampling. This design gave us three samples at each location, one feces, one shallow soil, and one deep soil for a total of thirty samples for each date. From each location, we had four experimental plating categories: deep soil, shallow soil, feces slurry, and feces ball. We divided feces into slurry and ball to examine differences between the interior and surface of feces in regards to our survey method. See below for feces slurry preparation.

### Culture of dictyostelids from soil and feces

We plated all samples within two days of collection on hay agar plates. We prepared hay agar plates following Douglas et al. (2013). For food bacteria, we used overnight cultures of single colonies of *Klebsiella pneumoniae* grown in sterile Luria Broth (10 gm tryptone, 5 gm Oxoid yeast extract, and 10 gm NaCl per liter DDH_2_O). *K. pneumoniae* was obtained from the *Dictyostelium* Stock Center (http://dictybase.org/StockCenter/StockCenter.html). To prepare the soil and feces for plating, we weighed five grams of soil or feces from each sample and placed it in a sterile 50 ml Falcon tube with sterile deionized water up to 50 ml. We dispersed the soil and feces in water by vortexing, then pipetted 300 μl of soil or feces slurry, per hay agar plate. We plated ten hay agar plates for each sample: five with 200 μl added overnight culture of food bacteria and five with no added food bacteria but with 200 μl Luria Broth added as control. Plating with and without added food bacteria allows us to germinate as many spores as possible present in the soil and feces samples in case suitable food bacteria for *D. discoideum* was not present or did not multiply under our growth conditions. After the liquid on each plate was absorbed (leaving the plates open in a biological safety cabinet for about 2–5 min), we added 6–12 activated charcoal pieces (Mars Fishcare Inc.) weighing about 100 milligrams. Activated charcoal can absorb a gaseous repellent released by culminating fruiting bodies aiding in their collection (Bonner and Dodd, 1962). Additionally, we placed one feces ball on each of five hay plates for each site. The feces ball was placed in a small indentation in the agar in the center of the hay plate with no additional bacteria added. We left the hay plates at room temperature (21°C) and checked them for growth of *D. discoideum* using a dissecting microscope for 6–10 days after plating. *D. discoideum* are readily and easily identifiable by their unique morphology compared to other Dictyostelids. We counted the number of distinct groups of fruiting bodies as areas positive for *D. discoideum* for each type of soil and feces (Supplementary Table 2). However, *D. discoideum* spores from the same soil sample could have come from the same fruiting body during the original collection process. Therefore, if more than one positive area is present on a test plate from the same soil sample, each area has the potential to be genetically different but may also be genetically the same.

### Bacterial presence or absence in *D. discoideum* fruiting bodies

We found one to three distinct groupings of *D. discoideum* fruiting bodies on the positive hay plates, and we tested the sorus from a single fruiting body from each grouping to see if they contained bacteria. To do this, we used a sterile pipette tip to collect the sorus from a single fruiting body while viewing under a dissecting microscope. We placed the sorus in a 1.5 ml Eppendorf tube containing 50 μl of sterile non-nutrient buffer (2.25 g KH_2_PO_4_ and 0.67 g K_2_HPO_4_ per liter DDH_2_O). Next, we vortexed the Eppendorf tube to disperse the fruiting body sorus contents. We then plated 10 μl of the sorus suspension on each of two SM/5 nutrient agar plates (2 g glucose, 2 g Oxoid bactopeptone, 2 g Oxoid yeast extract, 0.2 g MgSO_4_, 1.9 g KH_2_PO_4_, 1 g K_2_HPO_4_, and 15.5 g agar per liter DDH_2_O) either in association with *K. pneumonia* or in non-nutrient buffer at room temperature (21°C). This technique will reveal aerobic bacteria carried in *D. discoideum* sori that can be cultured, but not unculturable or anaerobic bacteria. We then sequenced 198 16S rRNA bacterial isolates from 95 fruiting body sori after streaking them to pick up a single clonal isolate (Supplementary Table 3). Twenty-four of the sequenced bacteria isolates had the same 16S rRNA identity as our food bacteria *K. pneumoniae*. We removed these bacteria sequences to eliminate any chance they were introduced during our isolation procedure and were not present in the soil or feces samples. We used the remaining 174 sequences in all subsequent calculations.

### PCR amplification, bacterial identification, and diversity analyses

We used the procedure outlined in “Identifying Unknown Bacteria using Biochemical and Molecular Methods” (www.nslc.wustl.edu/elgin/genomics/Bio3055/IdUnknBacteria06.pdf) to prepare template bacterial DNA for sequencing with one modification to the procedure. Instead of collecting bacteria grown in overnight liquid cultures, we collected a small amount of stationary phase bacteria clonally grown on a nutrient agar plate and prepared a suspension in water. The PCR amplification was done using a Gene Amp kit from Applied Biosystems (Roche). We used forward sequence 5′-CGG CCC AGA CTC CTA CGG GAG GCA GCA G-3′ and reverse sequence 5′-GCG TGG ACT ACC AGG GTA TCT AAT CC-3′ as primers to amplify about 460 bp of the 16S ribosomal RNA gene. The PCR fragments we generated were Sanger sequenced at GeneWiz (www.genewiz.com; South Plainfield New Jersey). Sequences were cleaned using Geneious 6.0 (http://www.geneious.com; Kearse et al., 2012), then aligned and clustered into OTUs (97% similarity) and identified with mothur (Schloss et al., 2009). We aligned sequences and taxonomically classified them using release 128 of the SILVA rRNA database (https://www.arb-silva.de/documentation/release-128/). We generated rarefaction curves and species richness estimates following the guidelines of Gotelli and Colwell (2011), and ran AMOVA (Analysis of Molecular Variance) analysis in mothur. We pooled the deer feces slurry and ball samples for these analyses because very few bacteria associated with *D. discoideum* were isolated from the ball samples.

### Phylogeny construction

We aligned unique haplotypes of our cleaned sequences in Geneious 6.0, along with representative taxa of each major bacterial clade. We reconstructed the phylogeny using a maximum likelihood analysis in Mega 6 (Tamura et al., 2013). We used a general time-reversible model of sequence evolution and rooted the tree at the midpoint. Statistical support was generated using 1,000 bootstrap replicates. We display the phylogeny as a cladogram with edibility and sampling data mapped on using ITOL (Letunic and Bork, 2006). The full phylogeny with bootstrap values is included in the Supplementary Materials.

### Edibility assay

We tested bacterial isolates for edibility against four wild *D. discoideum* strains collected from this survey that were unassociated with bacteria. For this assay, we define edible bacteria as those bacteria with the capacity to support growth of *D. discoideum*. We used 159 bacteria isolates rather than all 174 bacteria species from our survey because fifteen grew too poorly on plates to assay. We chose two *D. discoideum* strains isolated from soil (4S 6.1 and 7S 4.1) and two *D. discoideum* strains isolated from feces (14P 2.2 and 18P 7.1). For the assay, we grew these four *D. discoideum* strains from spores on SM/5 nutrient agar plates supplemented with *K. pneumoniae* as our food bacteria at room temperature (21°C). We also grew each test bacterial isolate on SM/5 nutrient agar plates under the same conditions. Using a sterile inoculating loop, we first gathered a small amount of stationary phase test bacteria and then gathered spores from one of the test *D. discoideum* using the same loop. We streaked the bacteria/spore mixture densely across one quadrant of a subdivided SM/5 nutrient agar plate. We repeated the same procedure with different *D. discoideum* isolates across the other three quadrants. See Supplementary Figure 1 for a cartoon schematic of the assay. After 1 week, we scored and tabulated the plates for edibility using the following qualitative categories: excellent (no bacteria present and abundant fruiting bodies), good (few bacteria present and many fruiting bodies), poor (many bacteria present and few fruiting bodies), inedible (abundant bacteria present and no fruiting bodies). See Figure 7 for cartoon and representative images of edibility assay. We used Pearson's Chi-square goodness of fit to determine if our sample data are consistent with a hypothesized distribution of equal proportions as our null hypothesis, or of dissimilar distributions between feces and soil environments. We used software available from http://quantpsy.org/chisq/chisq.htm to calculate the chi-square test (Preacher, 2001).

### Persistence assay

We plated spores from one naïve clone unassociated with bacteria (QS9) individually with eighteen different bacteria genera to test for persistence through multiple *D. discoideum* social cycle rounds without additional bacteria. Seventeen genera were isolated from this screen. The bacteria genera tested were *Achromobacter, Ancylobacter, Burkholderia, Comamonas, Escherichia, Flavobacterium, Oxalicibacterium, Paenibacillus, Pandoraea, Pseudomonas, Rahnella, Rhizobium, Serratia, Shinella, Staphylococcus, Stenotrophomonas, Variovorax*. We used the lab food bacteria *Klebsiella pneumoniae* as a negative (no persistence) control for a total of eighteen genera. We streaked the bacteria onto SM/5 plates from frozen stocks and grew to stationary phase at 21°C. We prepared bacterial suspensions in non-nutrient buffer at an OD_600_ of 1.5. To set up the assay, we collected spores from fruiting bodies under a dissecting scope by touching the top of a sorus using a sterile pipette tip. The spores were placed into sterile non-nutrient buffer. We determined spore density using a hemacytometer and a light microscope. We plated 2 × 10^5^ spores and 200 μl of each of the eighteen test bacteria onto SM/5 nutrient agar plates. This is the initial plating. After the initial plating, all subsequent transfers were done bacteria-free meaning spores were collected from each test plate in non-nutrient buffer and plated onto nutrient agar as above without adding additional test bacteria. Bacteria growth and the eventual formation of fruiting bodies would only be able to occur in the test rounds if bacteria persisted in the spores from the initial plating with the test bacteria genera. We grew all rounds at room temperature (21°C) until fruiting bodies formed. Next, for each round we placed ten individual spore masses (sori) from randomly selected fruiting bodies onto SM/5 nutrient agar (spot test). We recorded bacterial growth and fruiting body formation in the individual test spots of spore masses after 5 days at room temperature (21°C).

## Results

**Figure 2:**
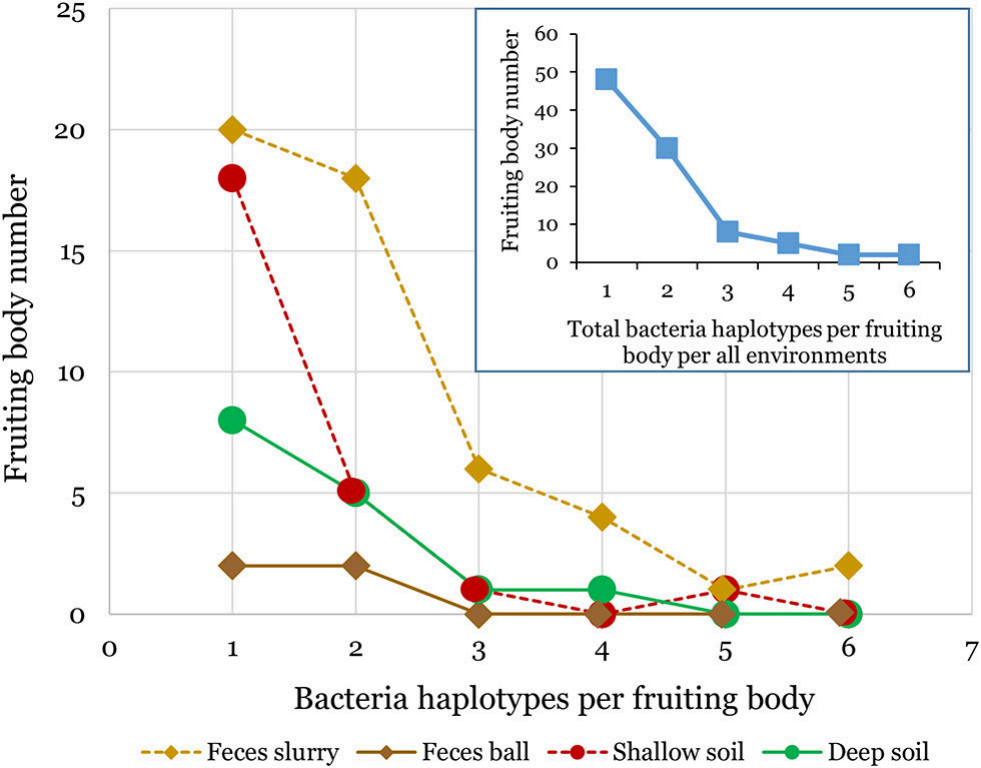
Multiple bacteria isolates can transiently persist within individual *D. discoideum* fruiting bodies. We found bacteria associated with about one-third of the *D. discoideum* fruiting bodies isolated in this survey. Each individual fruiting body sorus positive for bacteria presence collected from either soil or feces environments contained from one to six bacteria isolates as shown in the above line graphs. The inset line graph quantifies the total number of bacteria isolates per fruiting body we isolated from all environments combined.

We found bacteria associated with 95 of the 254 *D. discoideum* sori isolated from this survey. The sorus is the spore-containing area located at the top of the fruiting body. The other 161 inspected sori contained no culturable bacteria. We isolated *D. discoideum* at all twenty locations from at least one type of soil or feces sample using either added or no added food bacteria (Figure 1; Supplementary Table 2). In total, we clonally isolated 174 culturable bacteria isolates (Supplementary Table 3). We found that one to six genetically distinct bacteria isolates can transiently persist through a social cycle in a single sorus isolated from wild *D. discoideum* (Figure 2). Although a fruiting body sorus may contain up to six bacteria isolates, we found the majority (82.1%) contain one or two bacteria isolates per fruiting body sorus.

**Figure 3:**
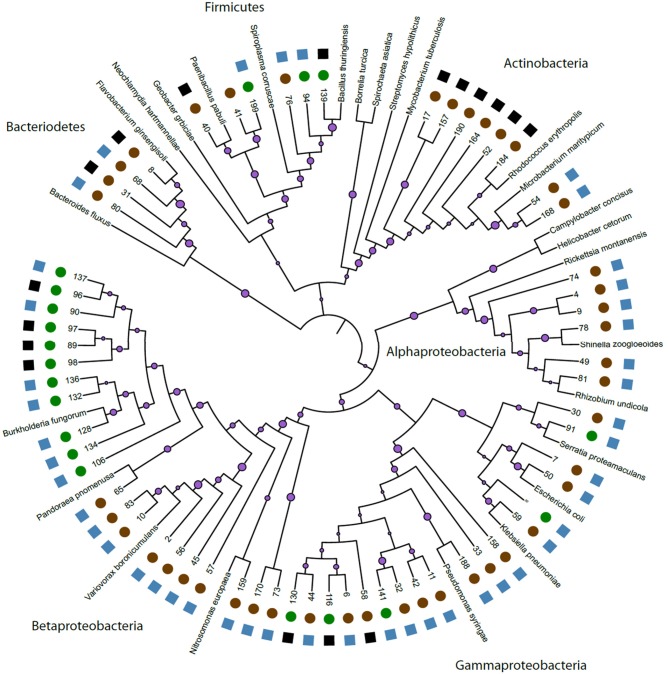
Phylogeny of unique bacteria haplotypes. We created a phylogeny of unique bacteria haplotypes found in association with *D. discoideum*. We anchored this phylogeny with 26 named and sequenced bacteria. On the individual branches, we have purple circles representing bootstrap support from 1,000 bootstrap replicates with larger circle size indicating greater support. Bootstrap numbers for each branch length can be found in Supplementary Figure 2. The numbers at the end of the branches represent the bacteria we isolated during this survey. See Supplementary Table 3 for further details. On the outside edge of the figure, we used circles to represent bacteria from our two environments: feces (brown) and soil (green). We used squares to represent edibility: edible (blue) and inedible (black) for each unique haplotype. Edibility for some bacteria genera had both inedible and edible bacteria and the blue or black edibility symbols square shown here represent a single unique bacterium in that genus.

**Figure 4:**
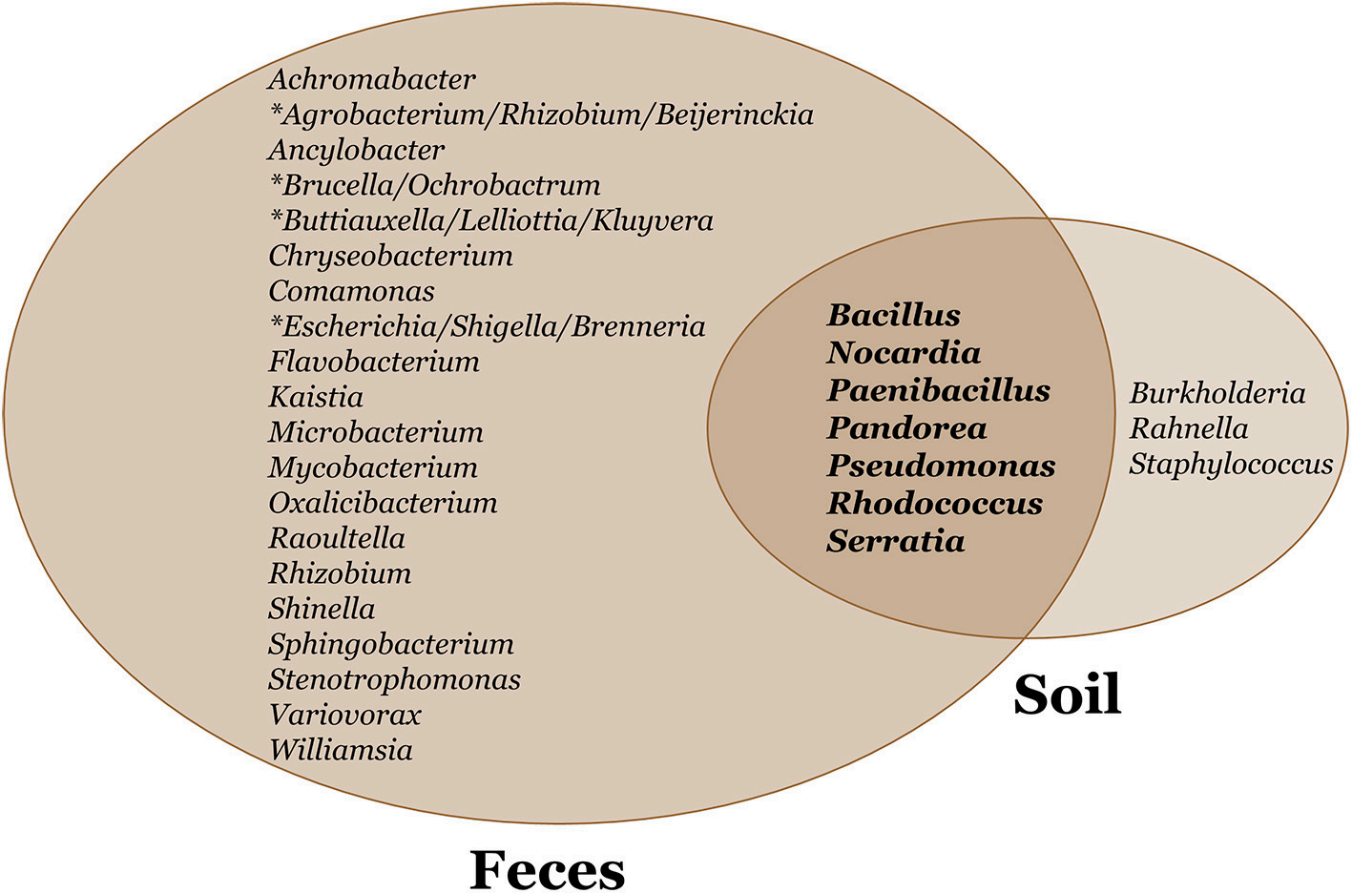
Venn diagram of soil and feces bacteria genera associated with *D. discoideum*. We found seven bacteria genera overlap between soil and feces environments. We identified about twice as many culturable bacteria genera associated with *D. discoideum* isolated from feces compared to soil environments. A 460 bp PCR fragment generated from 16S rRNA primers was sequenced to identify these bacteria genera. The asterisk signifies bacteria genera of equal sequence identity.

We constructed a phylogeny of bacteria using unique haplotypes from 16S rRNA sequences (Figure 3). These haplotypes are located in four bacteria phyla: Actinobacteria, Bacteriodetes, Firmicutes, and Proteobacteria, the latter represented by three classes (Alpha, Beta, and Gamma). We found the majority (71.4%) of these unique bacterial haplotypes are Proteobacteria and these haplotypes are almost all edible (84.4%). Additionally, the bacteria haplotypes from Actinobacteria, Alphaproteobacteria, and Bacteriodetes were isolated only from *D. discoideum* originating from feces environments. There were about twice as many culturable bacteria genera associated with *D. discoideum* isolated from feces compared to soil environments. Seven genera overlap between the soil and feces environments (Figure 4), and these account for 63.8% of bacterial isolates from this survey.

**Figure 5:**
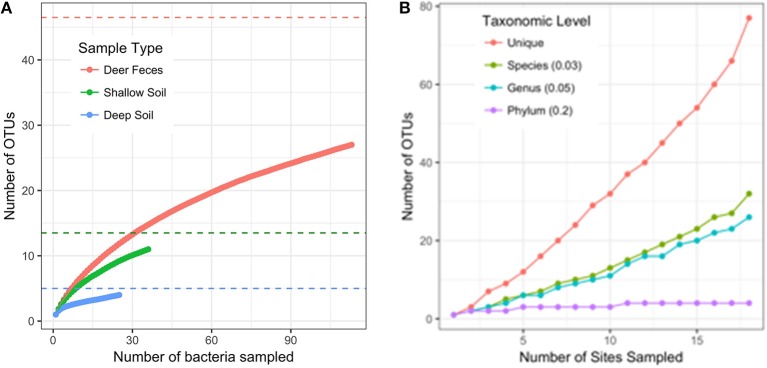
Rarefaction curves**. (A)** Rarefaction curve for the number of bacterial isolates sampled separated by sample type (intact deer feces and feces slurry samples are combined into one category). OTUs are defined with a distance of 0.03. Dashed lines are the Chao1 estimates of species richness for the sample type with the corresponding color. **(B)** Rarefaction curve for the number of sites sampled. OTUs are defined at different taxonomic levels following the distance cutoffs recommend by Schloss and Handelsman, 2005. The “unique” category counts every non-identical sequence as a single OTU.

When 16S rRNA sequences were clustered into OTUs at 97% similarity, we found the diversity of bacteria was highest in deer feces (27 OTUs), and lower in both the shallow (11 OTUs) and deep soil (4 OTUs) samples. Our experimental design included adding food bacteria (*K. pneumoniae*) to half of the selection plates. We found the addition of *K. pneumoniae* had no effect on the diversity of bacteria we collected from *D. discoideum* (AMOVA, *F*_(1, 172)_ = 1.04, *p* = 0.346; with Kp = 25 OTUs, without Kp = 22 OTUs). Since each sample type varied in the number of bacteria we were able to culture, we used rarefaction curves and species richness estimates to compare the diversity between the sample types. None of the rarefaction curves reached saturation, indicating we did not fully sample the diversity in any sample type (Figure 5A). However, the Chao1 species richness estimates suggest that the total number of OTUs associated with *D. discoideum* at Mountain Lake is low and could be fully sampled with moderately more effort. The deer feces had a Chao1 estimated richness of 46.5 bacterial associates (95% CI of 32.2–99.9) while the shallow and deep soil were estimated to have 13.5 (11.3–28) and 5 (4.1–17.3) bacterial associates, respectively. We also assessed how well the number of sites we sampled captured the bacterial diversity since individual-based rarefaction can overestimate species richness when species distribution is patchy (Gotelli and Colwell, 2011), which is likely for *Dictyostelium* and its associated bacteria. Rarefaction curves at different taxonomic levels indicate we did not fully sample bacterial diversity except at the phylum level (Figure 5B). As indicated by the sample type rarefaction, most of this undiscovered diversity is likely to be in deer feces.

**Figure 6:**
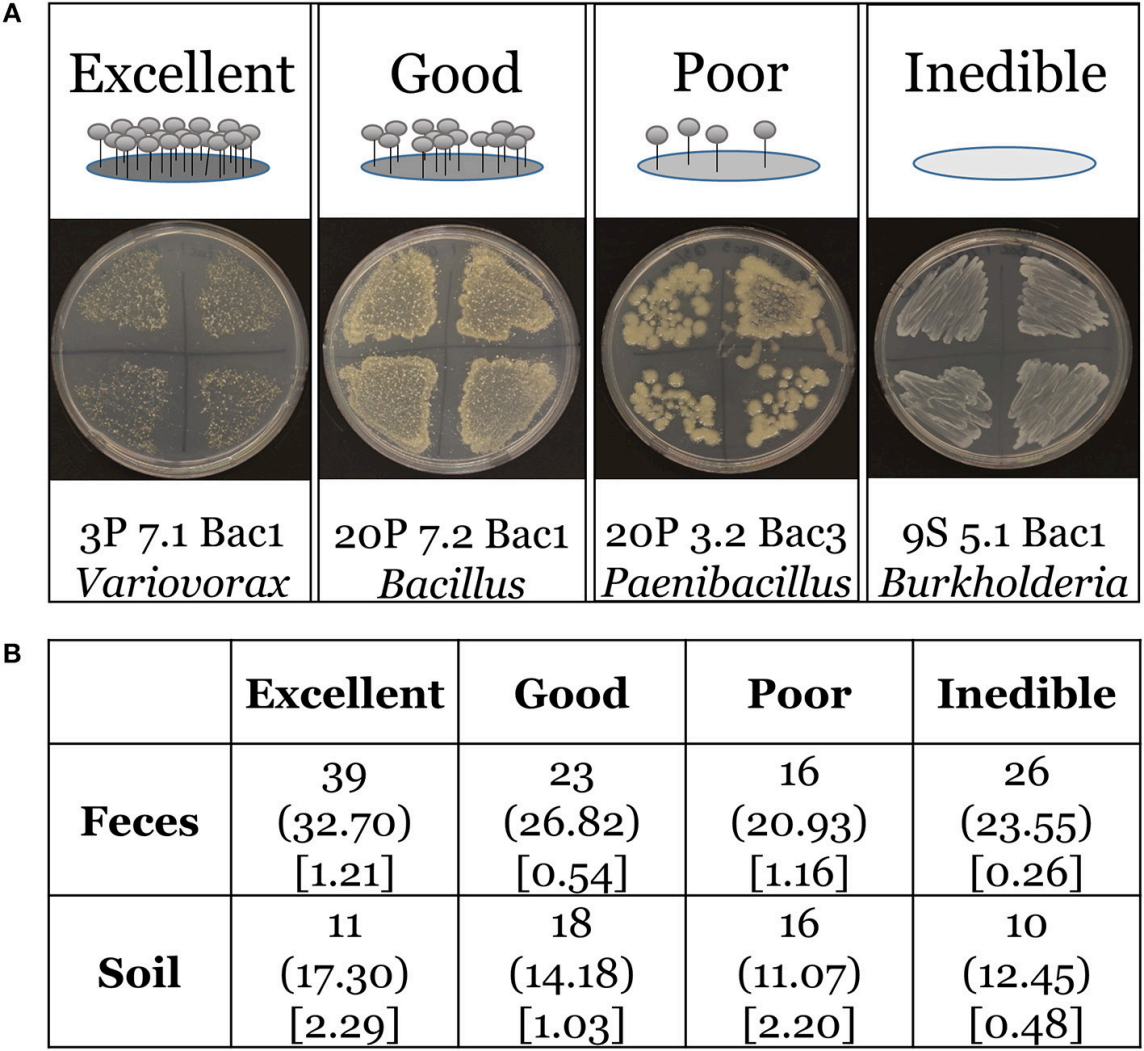
Edibility assay rubric and Chi-square contingency table.**(A)** Top panel includes cartoon examples of our scoring rubric for bacteria edibility by wild *D. discoideum*: excellent (no bacteria present and abundant fruiting bodies), good (few bacteria present and many fruiting bodies), poor (many bacteria present and few fruiting bodies), inedible (abundant bacteria present and no fruiting bodies). Bottom panel includes representative images of bacteria and fruiting bodies on nutrient agar plates corresponding to each category from the assay. **(B)** Chi-square contingency table. The contingency table provides the following information for the edibility data: the top line is the observed total for each edibility type, the middle line in parentheses is the expected total for each edibility type, and the bottom line in brackets is the chi-square statistic for each edibility type.

We asked if the distribution of our four edibility types is the same in both feces and soil environments. *D. discoideum* amoebae generally lyse and digest phagocytized bacteria very quickly (Clarke and Maddera, 2006). Yet we found numerous bacteria persisting through the multicellular stage, which is triggered by starvation. Are these bacteria persisting because they were inedible and could not be broken down? We scored and tabulated edibility using four categories: excellent, good, poor, and inedible (Figure 6A). We found 75.2% and 81.8% of *D. discoideum*-associated bacteria from feces and soil habitats, respectively are edible with a range from excellent to poor. Additionally, we asked if there were any differences between soil-collected and feces-collected bacteria in the degree of edibility. Our null hypothesis is that no relationship exists between the distributions of edibility categorical types from the feces and soil sets in the population. We performed Pearson's chi-square test and rejected this hypothesis. The distributions of bacterial edibility types that associate with *D. discoideum* differ significantly from expected between soil and feces habitats though not strongly (chi-square = 9.1713; df = 3; *p* = 0.027; Figure 6B). The main driver is the larger proportions of excellent edible type and inedible type bacteria found in feces compared to soil.

**Figure 7:**
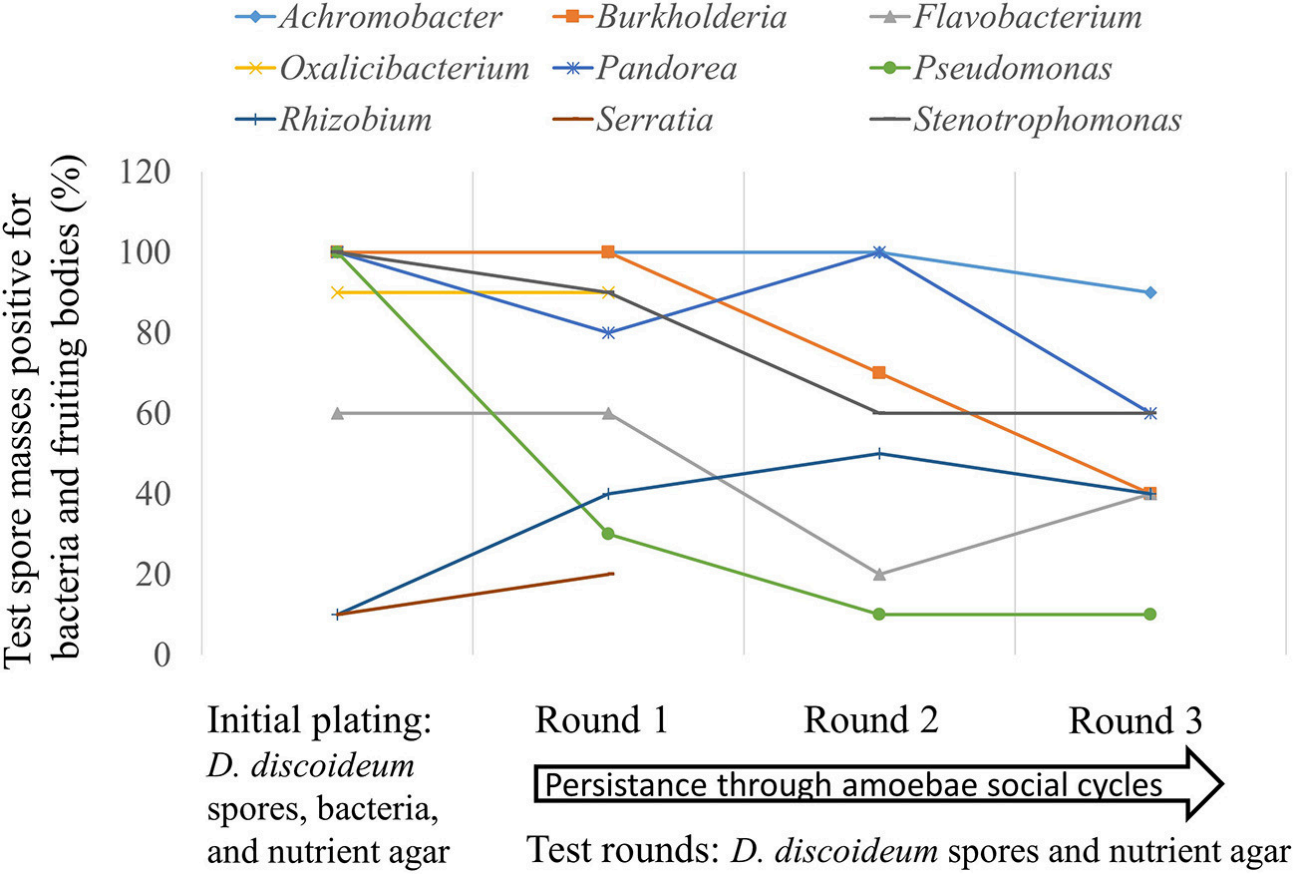
Some bacteria genera are able to evade phagocytosis and persistently associate with *Dictyostelium* amoebae through multiple social cycles. We plated spores from one naïve non-farmer *D. discoideum* clone individually with 17 different bacteria genera isolated from this screen and the lab food bacteria *Klebsiella pneumoniae* as control (initial plating). After fruiting bodies formed, we serially passaged *D. discoideum* spores from each test bacteria for three rounds with no additional bacteria (test rounds). We tested ten random individual spore masses from fruiting bodies formed after completion of the social cycle for the presence of bacteria and fruiting bodies on nutrient agar. We did this after each round including the initial plating. Bacteria growth and the eventual formation of fruiting bodies would only be able to occur in the test rounds if bacteria persisted in the spores from the initial plating with the test bacteria genera. Nine of the eighteen bacteria genera evaded phagocytosis and were able to persist through one round of *D. discoideum* fruiting body formation on nutrient agar without additional food bacteria added; seven of the eighteen persisted through three social cycle rounds. The nine genera tested that did not persist are: *Ancylobacter, Comamonas, Escherichia, Klebsiella, Paenibacillus, Rahnella, Shinella, Staphylococcus*, and *Variovorax*.

Avoidance of digestion by bacteria after phagocytosis is thought to have first evolved as a method to resist predatory amoeba (Cosson and Soldati, 2008). We tested seventeen bacteria genera from our screen with the lab food bacteria *K. pneumoniae* as control to determine if these edible bacteria were able to persist through multiple social cycles (Figure 7). Each tested bacteria was the only food source for the *D. discoideum* amoebae so if persistence occurred the bacteria would be partially evading phagocytosis. We found nine out of 18 bacteria tested persisted through the first round and seven out of 18 through three social cycle rounds (Figure 7).

## Discussion

The extent of natural variation in *D. discoideum* relationships with soil bacteria has been largely unexplored. In this study, we investigated the range of culturable aerobic bacterial associations with *D. discoideum* fruiting bodies in a natural environment by conducting a survey of forest soil and deer feces at Mountain Lake Biological Station. *D. discoideum* are predators of bacteria and were previously thought to be bacteria-free during all life stages (Raper, 1937) until we reported some *D. discoideum* maintain a symbiotic relationship with a handful of *Burkholderia* species (Brock et al., 2011; DiSalvo et al., 2015). In this report, we demonstrate that newly isolated wild *D. discoideum* fruiting bodies are far from bacteria-free and are able to transiently carry a broad variety of bacteria throughout their life history. We also establish that many of the bacteria are able to evade phagocytosis and persist through multiple social cycle rounds lab conditions.

Overall, our collection strategy yielded similar numbers of *D. discoideum* clones from feces compared to soil environments. We isolated numerous bacteria from about one-third of these *D. discoideum* clones after terminal differentiation when fruiting bodies are formed. Edible bacteria make up the majority of the bacteria we collected (85% of feces and 81% of soil) and the high degree of edibility is in agreement with previous early reports that the type-clone of *D. discoideum* could feed on a wide range of bacterial species (Raper, 1937; Raper and Smith, 1939). The soil samples seem to be fairly poor sources of *D. discoideum*-associated bacteria based on the lower diversity of culturable bacteria in shallow soil (11 OTUs) and deep soil (4 OTUs) compared to deer feces (27 OTUs). Despite being extremely bacteria-rich (Fierer, 2017), soil may have a scarcity of bacteria that serve as prey or are able to persist with *D. discoideum*. Deer feces, another environment densely colonized by bacteria, harbored more bacteria that could associate with *D. discoideum*, suggesting the type of habitat *D. discoideum* grows in likely affects which bacteria are available for uptake. In addition, the relatively few bacteria (Chao1 estimates of 5–47 culturable species) found within *D. discoideum* living in such bacteria-rich environments suggests that the association may be selective rather than being either promiscuous or haphazard. It is also important to note that we focused here on culturable aerobic bacteria in order to test the edibility and persistence of the bacteria collected. However, there are likely to be other bacteria associated with *D. discoideum* that cannot grow on the media we used, and a culture-free survey is necessary to get a complete picture of the *D. discoideum* microbiome.

The many bacteria we isolated associated with our wild *D. discoideum* isolates may or may not be residing inside the spores. These bacteria could be associating with *D. discoideum* in some other fashion. We have previously established that some wild *D. discoideum* known as farmers facultatively associate with certain bacteria, that these bacteria are located inside spores, and that they can be vertically transmitted to the next generation (Brock et al., 2011; DiSalvo et al., 2015). In this study we found several bacteria genera were able to persist through multiple social cycle rounds suggesting wherever these bacteria are located they are able to be transmitted to the next generation in some fashion. However, spot test percentages were lower in the persistence assay than we have previously shown with symbiotic *Burkholderia* sp. nov. in *D. discoideum* (DiSalvo et al., 2015), so carriage in the spores or fidelity of transmission of the tested bacteria genera may be less than we found for our symbiotic *Burkholderia*. Where bacteria from this survey are located within the *D. discoideum* fruiting bodies will need to be determined by future research, but it seems likely that at least a portion of them could be persisting inside the spores.

Non-digestion (persistence) of bacteria in hosts can be aided by manipulation of the host immune response, and bacteria have developed an arsenal of diverse mechanisms to do this (Sansonetti and Di Santo, 2007; Ribet and Cossart, 2015). Secretion systems are commonly used by bacteria to secrete effectors into a host cell after engulfment (Green and Mecsas, 2016). These secreted effectors facilitate escape of the bacteria from the phagosome or block phagosome fusion to the lysosome preventing bacterial cell death thereby creating a new niche in the host. Proteobacteria are particularly rich in secretion systems (T1SS, T2SS, T3SS, T4SS, T5aSS, T5bSS, T5cSS, and T6SS) having more different types of secretion systems than all other phyla (Abby et al., 2016). The majority of bacteria we isolated in total were in the Alpha, Beta, and Gamma classes of Proteobacteria and this was also true of the type bacteria that persisted through multiple social rounds of growth. A similar abundance of Proteobacteria classes was found among digestion-resistant bacteria in surveys of marine and fresh water ciliates, another group of unicellular eukaryotes (Pucciarelli et al., 2015; Gong et al., 2016). Gong et al. (2016) suggested bacteria with type IV and VI secretion systems (T4SS and T6SS) as having a possible role in promoting these marine protist/bacteria associations. Both type IV and VI secretion systems transport proteins and effector molecules into eukaryotic cells and have been implicated in suppressing immunity and exporting virulence factors (Cascales and Christie, 2003; Bingle et al., 2008). Type VI secretion systems have also been shown to have non-pathogenic roles mediating symbiotic relationships between bacteria and eukaryotes as well (Parsons and Heffron, 2005; Chow and Mazmanian, 2010; Jani and Cotter, 2010). However, Proteobacteria are often abundant in forest soil (Janssen, 2006), so we are not certain how over-represented they might be in our samples.

Secretion systems are also one of the mechanisms bacteria use to secrete effectors that directly target components of the innate immune system such as Toll-like receptors and Nod-like receptors (Reddick and Alto, 2014). When multicellular, *D. discoideum* contain a cell type known as sentinel cells that provides immune-like and detoxification functions (Chen et al., 2007; Brock et al., 2016a). Additionally, Chen et al. (2007) found a Toll/Interleukin-1 Receptor (TIR) domain protein that is necessary for sentinel cells to effectively target bacteria to allow clearance of the bacteria from the slug. We have previously shown that wild *D. discoideum* farmers have fewer sentinel cells compared to *D. discoideum* with no associated bacteria suggesting a relaxed immune response promoting symbiotic interactions through a reduction in the clearance of carried bacteria (Brock et al., 2016a). The bacteria we found associated in the sori of wild *D. discoideum* fruiting bodies may be persisting through a similar mechanism.

Amoebae are proposed as reservoir hosts for pathogenic bacteria (Molmeret et al., 2005; Scheid, 2014; Strassmann and Shu, 2017). In support, Benavides-Montaño and Vadyvaloo recently reported *Yersinia pestis* can survive in *Acanthamoeba castellanii* amoebae for prolonged periods by using the type three secretion system to inhibit phagocytosis (Benavides-Montaño and Vadyvaloo, 2017). Bacteria can increase in virulence even after short associations with eukaryotes leading to environmental and health consequences. One example reports enhanced virulence of *Mycobacterium avium* in macrophage and mouse models after growth in *A. castellanii* (Cirillo et al., 1997). Another recent example describes increased dispersal ability and amplified virulence of *Bordetella bronchiseptica* after growth in *D. discoideum* along with macrophage and mouse models (Taylor-Mulneix et al., 2017). Many of the bacterial genera we isolated in this study are known to have members implicated in causing disease, such as *Brucella* (Brucellosis), *Escherichia/Shigella* (urinary tract infections/diarrhea), *Nocardia* (Nocardiosis), and *Staphylococcus* (urinary tract infections), suggesting similar possibilities for health consequences.

Digestion of bacteria by amoebae should occur quickly after engulfment (Clarke and Maddera, 2006). However, our ability to isolate culturable bacteria from wild fruiting body spore masses formed after starvation suggests the possibility of some level of persistence and host manipulation. Amoebae/bacteria relationships are important because they can inform us about the interdependent relationships of bacteria to all eukaryotes (Gilbert et al., 2012). The vast range of eukaryote-bacteria interactions can affect development, immunity, and evolution (Rosenberg and Zilber-Rosenberg, 2016). Based on the data presented here, we find a more multifaceted relationship between *D. discoideum* and bacteria than just that of predator/prey. *D. discoideum* has already been utilized extensively as a model system to study host/pathogen relationships as detailed in a recent review (Bozzaro and Eichinger, 2011), and more recently to study symbiotic relationships (Brock et al., 2011, 2013, 2016b; DiSalvo et al., 2015). This system will provide an excellent platform to discover if bacteria persistence in wild *D. discoideum* amoebae can initiate either increased infectivity in other hosts or, alternatively, symbiosis. Our study shows that the range of bacterial associates, whether pathogenic, symbiotic, or haphazard, is much larger than has been appreciated.

## Data accessibility

The bacterial 16S rRNA gene sequences from this study have been deposited in the Genbank numbers are MK177283–MK177456.

## Author contributions

DB, DQ, and JS designed the experiments. FB and JG mapped and collected the samples. DB, UB, and TD performed the experiments. DB, JRG, TH, DQ, and JS analyzed the results and wrote the manuscript. All authors approved the final manuscript.

### Conflict of interest statement

The authors declare that the research was conducted in the absence of any commercial or financial relationships that could be construed as a potential conflict of interest.
