# Supplementary material for: Diversity of Free-Living Environmental Bacteria and Their Interactions With a Bactivorous Amoeba
Source: Front Cell Infect Microbiol. 2018 Nov 23;8:411. doi: 10.3389/fcimb.2018.00411 (PMC6266680; doi:10.3389/fcimb.2018.00411)
Supplement: Supplementary file 1 [file Data_Sheet_1.docx]

Supplementary Material

Article Title

Debra A Brock*, Tamara S. Haselkorn, Justine R. Garcia, Usman Bashir, Tracy E. Douglas, Jesse Galloway, Fisher Brodie, David C. Queller, Joan E. Strassmann

*** Correspondence:** Debra A. Brock: dbrock@wustl.edu

## Supplementary Figures

**
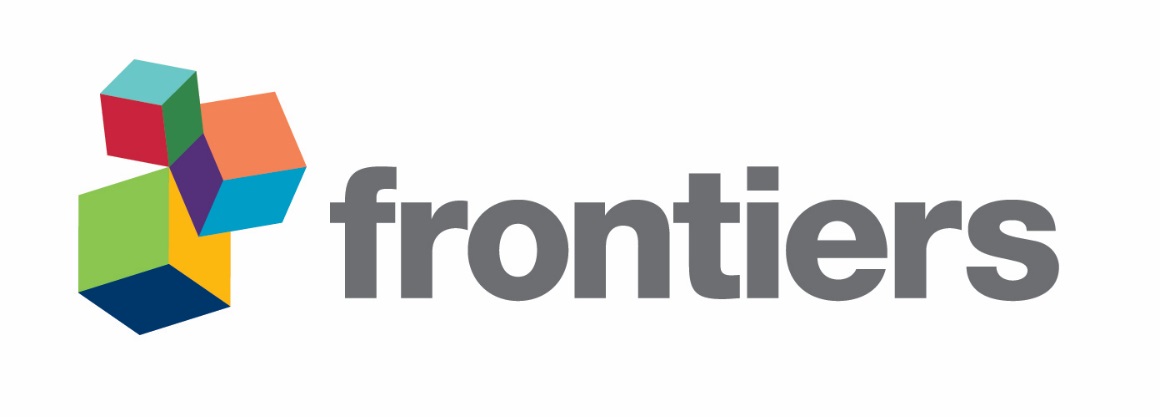
**

**Supplementary Figure 1.** **Cartoon schematic of edibility assay.**

We divided a SM/5 agar plate into four quadrants. To test a bacterium isolate for edibility, we gathered the test bacterium plus spores from one of the four test non-farmer wild *D. discoideum* using a sterile inoculating loop.  We used the loop to spread the bacteria and spores in the appropriate quadrant.  We placed the inoculated plates at 21°C for one week and then scored the test bacteria for edibility. The specific number identifier for the wild *D. discoideum* used in this assay is in parentheses in the cartoon figure below.


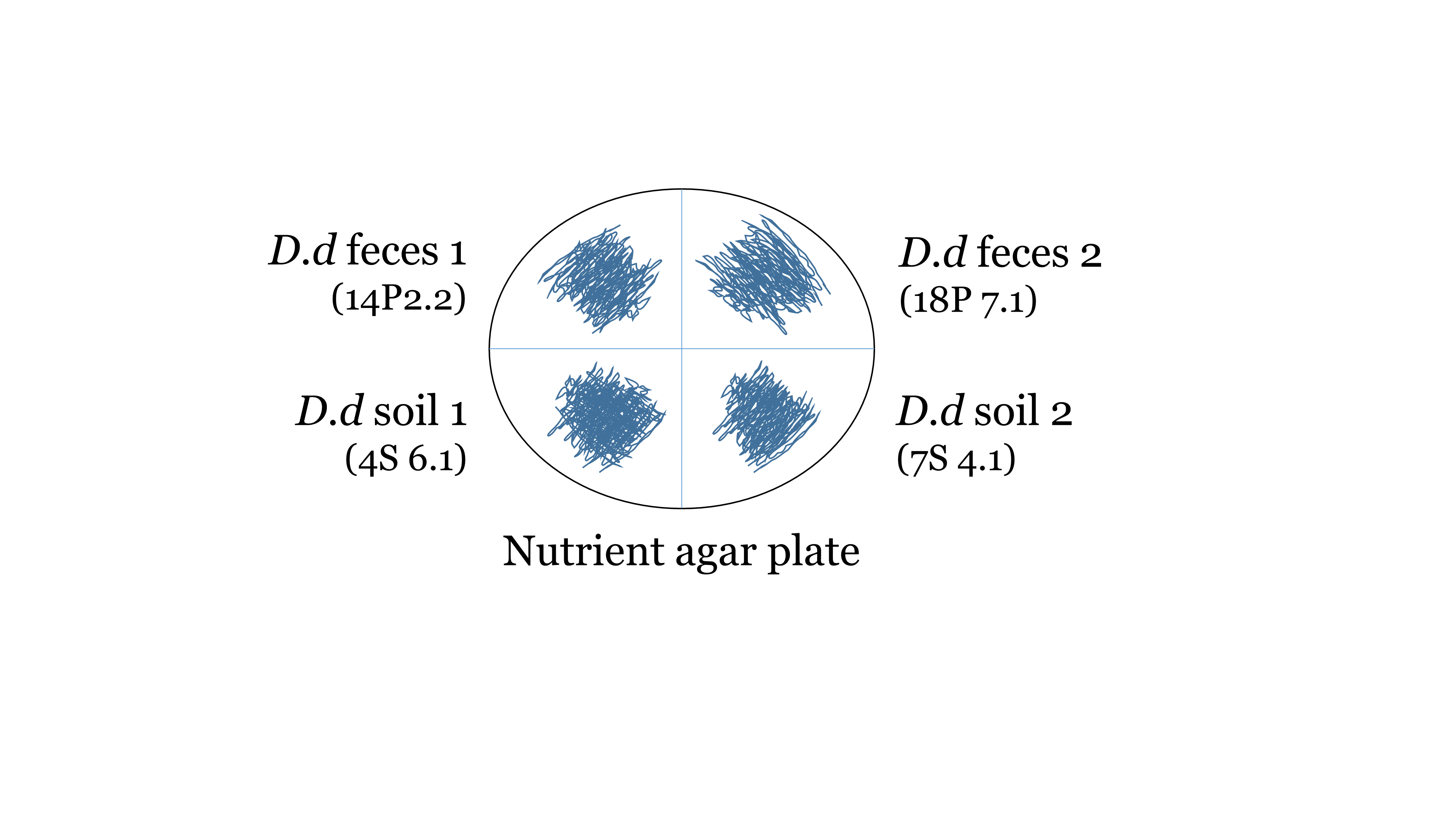


**Supplementary Figure 2. Tree of bacteria from soil samples.** We combined our unique bacteria haplotypes along with representative taxa of each major bacteria clade to construct a phylogeny based on maximum likelihood analysis. We used a general time-reversible model of sequence evolution rooting the tree at the midpoint. Statistical support was generated using 1000 bootstrap replicates with bootstrap values shown on the branches.


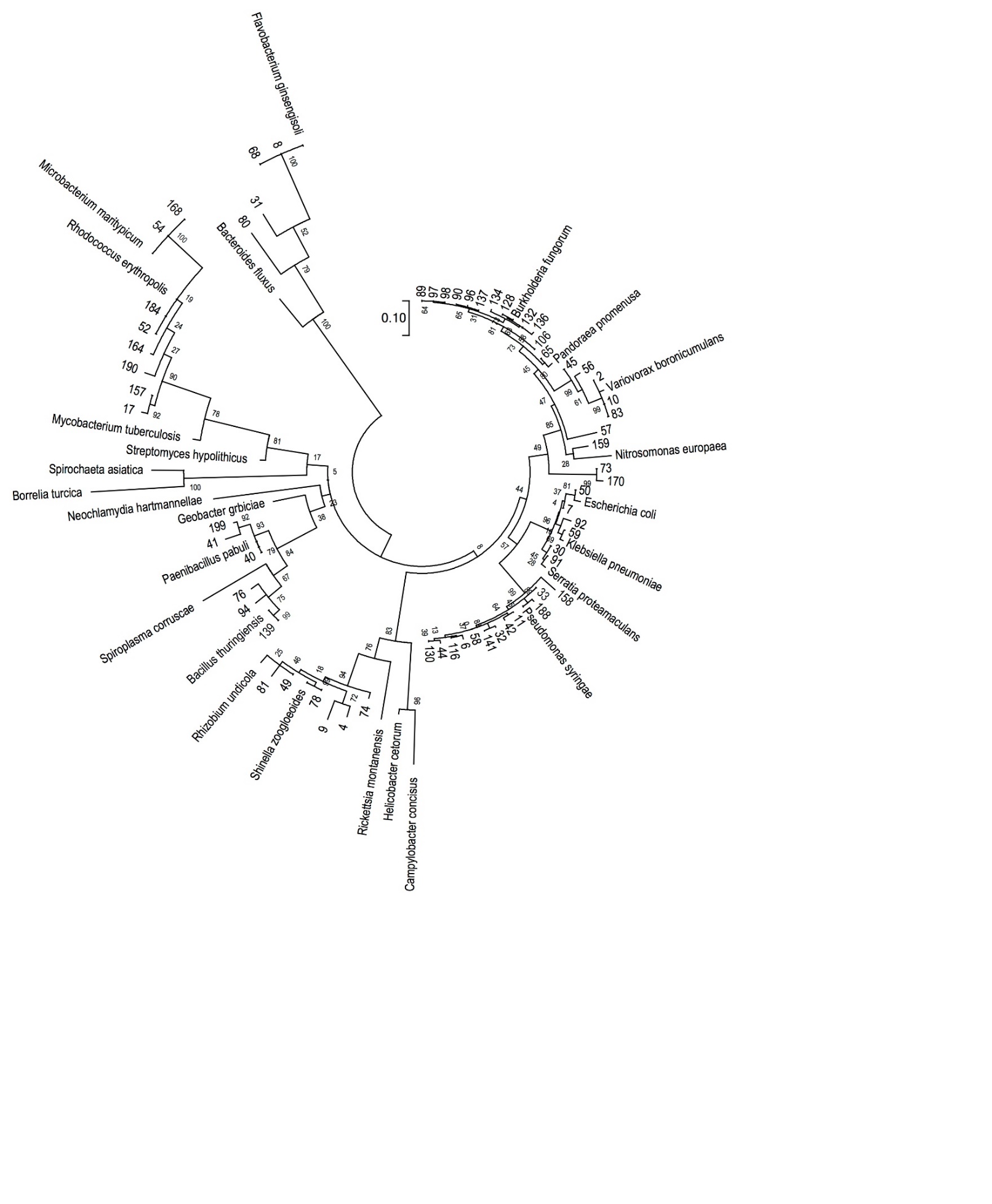


**Supplementary Table 1. GPS coordinates for individual collection sites*.*** We conducted a survey of bacteria associated with the eukaryote *Dictyostelium discoideum* on two separate dates in 2014 at Mt. Lake Biological Station, VA.

| **Collection** | **Collection Date** | **Sample Number** | **GPS Coordinates** | |
| --- | --- | --- | --- | --- |
| **Set 1** | 23 July 2014 | 1 | N 37.37538° | W 080.51962° |
| **Set 1** | 23 July 2014 | 2 | N 37.37541° | W 080.51955° |
| **Set 1** | 23 July 2014 | 3 | N 37.37537° | W 080.51944° |
| **Set 1** | 23 July 2014 | 4 | N 37.37571° | W 080.51913° |
| **Set 1** | 23 July 2014 | 5 | N 37.37612° | W 080.51908° |
| **Set 1** | 23 July 2014 | 6 | N 37.37611° | W 080.51913° |
| **Set 1** | 23 July 2014 | 7 | N 37.37613° | W 080.51920° |
| **Set 1** | 23 July 2014 | 8 | N 37.37614° | W 080.51923° |
| **Set 1** | 23 July 2014 | 9 | N 37.37613° | W 080.51926° |
| **Set 1** | 23 July 2014 | 10 | N 37.37617° | W 080.51931° |
| **Set 2** | 30 July 2014 | 11 | N 37.37659° | W 080.51953° |
| **Set 2** | 30 July 2014 | 12 | N 37.37680° | W 080.51956° |
| **Set 2** | 30 July 2014 | 13 | N 37.37697° | W 080.51961° |
| **Set 2** | 30 July 2014 | 14 | N 37.37680° | W 080.52007° |
| **Set 2** | 30 July 2014 | 15 | N 37.37674° | W 080.52050° |
| **Set 2** | 30 July 2014 | 16 | N 37.37734° | W 080.52112° |
| **Set 2** | 30 July 2014 | 17 | N 37.37820° | W 080.52170° |
| **Set 2** | 30 July 2014 | 18 | N 37.37712° | W 080.52200° |
| **Set 2** | 30 July 2014 | 19 | N 37.37696° | W 080.52190° |
| **Set 2** | 30 July 2014 | 20 | N 37.37682° | W 080.52381° |

**Supplementary Table 2. Positive collection locations for *D. discoideum***. Numbers represent the areas on an agar plate positive for *D. discoideum* fruiting bodies at each site and in each condition. A positive area for *D. discoideum* is defined as distinct separate groupings of fruiting bodies on the agar test plate. These areas may have grown from a single hatched spore thus different areas have the potential to be genetically different but may also be genetically the same. *Klebseilla pneumoniae* (*Kp*) is our food bacteria for *D. discoideum*.

|  | **Shallow soil** | | **Deep soil** | | **Feces slurry** | | **Feces ball** | |
| --- | --- | --- | --- | --- | --- | --- | --- | --- |
| **Location** | **no *Kp*** | **with *Kp*** | **no**  ***Kp*** | **with *Kp*** | **no *Kp*** | **with *Kp*** | **no *Kp*** |  |
| **1** | 0 | 0 | 1 | 0 | 0 | 0 | 0 |  |
| **2** | 2 | 3 | 0 | 2 | 0 | 0 | 0 |  |
| **3** | 1 | 4 | 0 | 3 | 3 | 2 | 0 |  |
| **4** | 1 | 1 | 0 | 0 | 0 | 0 | 0 |  |
| **5** | 9 | 11 | 0 | 0 | 6 | 0 | 0 |  |
| **6** | 0 | 0 | 10 | 13 | 0 | 0 | 0 |  |
| **7** | 4 | 7 | 1 | 0 | 1 | 5 | 0 |  |
| **8** | 0 | 0 | 3 | 0 | 0 | 0 | 0 |  |
| **9** | 3 | 5 | 0 | 0 | 0 | 0 | 0 |  |
| **10** | 0 | 0 | 0 | 0 | 8 | 0 | 0 |  |
| **11** | 0 | 0 | 0 | 0 | 5 | 7 | 0 |  |
| **12** | 0 | 0 | 0 | 1 | 0 | 1 | 0 |  |
| **13** | 0 | 0 | 0 | 0 | 3 | 1 | 9 |  |
| **14** | 0 | 1 | 0 | 0 | 13 | 9 | 9 |  |
| **15** | 0 | 2 | 0 | 0 | 0 | 0 | 0 |  |
| **16** | 2 | 2 | 0 | 0 | 0 | 0 | 0 |  |
| **17** | 0 | 2 | 0 | 8 | 0 | 1 | 0 |  |
| **18** | 0 | 0 | 0 | 0 | 12 | 10 | 0 |  |
| **19** | 0 | 4 | 0 | 0 | 0 | 6 | 0 |  |
| **20** | 3 | 12 | 0 | 0 | 9 | 11 | 2 |  |
| **Total** | 25 | 54 | 15 | 27 | 60 | 53 | 20 |  |

**Supplementary Table 3. 16S rDNA sequence affiliation in GenBank for bacteria transiently-associated with wild *Dictyostelium discoideum* with edibility data.** We isolated 174 bacteria transiently affiliated with *D. discoideum* collected from soil and feces samples at Mt. Lake Biological Station, VA. The samples are grouped by sample type: feces slurry, feces ball, shallow soil, and deep soil. We used the following naming rubric for bacteria isolated from wild collected *D. discoideum* sori found in the second column of this table. The first number represents the collection site (1-20). The second letter stands for sample type: shallow soil (S), deep soil (D), feces slurry (P) and feces ball (B). The next number indicates if the sample was plated with or without bacteria: 1-5 are no added bacteria; 6-10 are with added food bacteria. The last number indicates a *D. discoideum* fruiting body. For example, the first sample in the table below is 3P 7.1 Bac1. This sample came from the 3^rd^ collection location, was a feces slurry sample, was grown with added food bacteria, was from the first and/or only area containing fruiting bodies on that plate, and one or more bacteria ssp. was isolated from the sorus of this fruiting body. The third column contains edibility data. Edibility was scored as (E) excellent (all bacteria eaten and numerous fruiting bodies), (G) good (most bacteria eaten and many fruiting bodies), (P) poor (little bacteria eaten and few fruiting bodies), and (I) inedible (no fruiting bodies). The next six columns contain the 16S rRNA sequence identity match and information about that match. Many of our bacteria isolates had more than one top match with the same percent identity and alignment length. Included in this table is the first top match.

| **Sample Type** | **ID** | **Edibility** | **Template DNA #** | **Top Blast Hit** | **Subject ID** | **Percent Identity** | **Alignment Length** | **Mismatches** | **E-value** |
| --- | --- | --- | --- | --- | --- | --- | --- | --- | --- |
| Feces slurry | 3P 7.1_Bac1 | E | 1 | Variovorax boronicumulans strain NBRC 103145 16S rib... | NR_114214.1 | 99.757 | 411 | 1 | 0 |
| Feces slurry | 3P 9.1_Bac1 | E | 2 | Variovorax boronicumulans strain NBRC 103145 16S rib... | NR_114214.1 | 99.757 | 411 | 1 | 0 |
| Feces slurry | 3P 9.1_Bac2 | G | 3 | Paenibacillus pabuli strain NBRC 13638 16S ribosomal... | NR_113627.1 | 100 | 410 | 0 | 0 |
| Feces slurry | 5P 2.1_Bac1 | E | 4 | Ancylobacter abiegnus strain Z-0056 16S ribosomal RN... | NR_117472.1 | 100 | 386 | 0 | 0 |
| Feces slurry | 5P 3.1_Bac1 | I | 157 | Mycobacterium hackensackense strain 147-0552 16S rib... | NR_115184.1 | 99.753 | 405 | 0 | 0 |
| Feces slurry | 5P 3.1_Bac2 | E | 158 | Pseudomonas anguilliseptica strain S 1 16S ribosomal... | NR_029319.1 | 97.567 | 411 | 10 | 0 |
| Feces slurry | 5P 3.1_Bac3 | E | 159 | Oxalicibacterium horti strain NBRC 13594 16S ribosom... | NR_113620.1 | 99.027 | 411 | 4 | 0 |
| Feces slurry | 5P 3.1_Bac4 | G | 160 | Paenibacillus pabuli strain NBRC 13638 16S ribosomal... | NR_113627.1 | 100 | 410 | 0 | 0 |
| Feces slurry | 5P 5.1_Bac1 | E | 6 | Pseudomonas vranovensis strain 2B2 16S ribosomal RNA... | NR_043313.1 | 100 | 411 | 0 | 0 |
| Feces slurry | 5P 5.1_Bac2 | E | 7 | Raoultella planticola strain DSM 3069 16S ribosomal ... | NR_119214.1 | 100 | 411 | 0 | 0 |
| Feces slurry | 7P 1.1_Bac1 | P | 8 | Flavobacterium ginsengisoli strain DCY54 16S ribosom... | NR_109024.1 | 100 | 405 | 0 | 0 |
| Feces slurry | 7P 1.1_Bac2 | E | 9 | Kaistia terrae strain 5YN7-3 16S ribosomal RNA gene,... | NR_108222.1 | 100 | 386 | 0 | 0 |
| Feces slurry | 7P 1.1_Bac3 | E | 10 | Variovorax boronicumulans strain NBRC 103145 16S rib... | NR_114214.1 | 99.27 | 411 | 3 | 0 |
| Feces slurry | 7P 10.2_Bac1 | E | 11 | Pseudomonas endophytica strain BSTT44 16S ribosomal ... | NR_136473.1 | 99.757 | 411 | 1 | 0 |
| Feces slurry | 10P 4.2_Bac1 | G | 12 | Paenibacillus pabuli strain NBRC 13638 16S ribosomal... | NR_113627.1 | 100 | 410 | 0 | 0 |
| Feces slurry | 11P 2.1_Bac1 | Poor growth; not frozen; no edibility | 13 | Paenibacillus pabuli strain NBRC 13638 16S ribosomal... | NR_113627.1 | 100 | 410 | 0 | 0 |
| Feces slurry | 11P 3.1_Bac1 | Poor growth; not frozen; no edibility | 14 | Paenibacillus pabuli strain NBRC 13638 16S ribosomal... | NR_113627.1 | 100 | 384 | 0 | 0 |
| Feces slurry | 11P 4.1_Bac1 | I | 15 | Mycobacterium arabiense strain YIM 121001 16S riboso... | NR_109734.1 | 98.974 | 390 | 4 | 0 |
| Feces slurry | 11P 5.1_Bac1 | P | 16 | Paenibacillus pabuli strain NBRC 13638 16S ribosomal... | NR_113627.1 | 100 | 410 | 0 | 0 |
| Feces slurry | 11P 6.1_Bac1 | I | 17 | Mycobacterium arabiense strain YIM 121001 16S riboso... | NR_109734.1 | 98.974 | 390 | 4 | 0 |
| Feces slurry | 11P 7.1_Bac1 | P | 18 | Mycobacterium arabiense strain YIM 121001 16S riboso... | NR_109734.1 | 98.974 | 390 | 4 | 0 |
| Feces slurry | 11P 7.1_Bac2 | I | 163 | Kaistia terrae strain 5YN7-3 16S ribosomal RNA gene,... | NR_108222.1 | 100 | 358 | 0 | 0 |
| Feces slurry | 11P 8.2_Bac1 | I | 19 | Mycobacterium arabiense strain YIM 121001 16S riboso... | NR_109734.1 | 98.974 | 390 | 4 | 0 |
| Feces slurry | 11P 8.2_Bac2 | I | 20 | Paenibacillus pabuli strain NBRC 13638 16S ribosomal... | NR_113627.1 | 100 | 410 | 0 | 0 |
| Feces slurry | 11P 8.3_Bac1 | G | 21 | Paenibacillus pabuli strain NBRC 13638 16S ribosomal... | NR_113627.1 | 100 | 410 | 0 | 0 |
| Feces slurry | 11P 8.3_Bac2 | I | 22 | Pseudomonas migulae strain NBRC 103157 16S ribosomal... | NR_114223.1 | 99.513 | 411 | 2 | 0 |
| Feces slurry | 11P 9.1_Bac1 | Poor growth; not frozen; no edibility | 23 | Paenibacillus pabuli strain NBRC 13638 16S ribosomal... | NR_113627.1 | 100 | 401 | 0 | 0 |
| Feces slurry | 11P 9.1_Bac2 | I | 24 | Nocardia coeliaca strain DSM 44595 16S ribosomal RNA... | NR_104776.1 | 100 | 389 | 0 | 0 |
| Feces slurry | 13P 3.1_Bac1 | P | 25 | Paenibacillus pabuli strain NBRC 13638 16S ribosomal... | NR_113627.1 | 100 | 410 | 0 | 0 |
| Feces slurry | 13P 3.1_Bac2 | G | 26 | Paenibacillus pabuli strain NBRC 13638 16S ribosomal... | NR_113627.1 | 100 | 410 | 0 | 0 |
| Feces slurry | 13P 4.1_Bac1 | I | 164 | Rhodococcus globerulus strain DSM 43954 16S ribosoma... | NR_118617.1 | 100 | 390 | 0 | 0 |
| Feces slurry | 13P 4.1_Bac2 | P | 165 | Paenibacillus pabuli strain NBRC 13638 16S ribosomal... | NR_113627.1 | 100 | 407 | 0 | 0 |
| Feces slurry | 13P 8.1_Bac1 | I | 28 | Nocardia coeliaca strain DSM 44595 16S ribosomal RNA... | NR_104776.1 | 100 | 390 | 0 | 0 |
| Feces slurry | 14P 4.1_Bac1 | I | 29 | Nocardia coeliaca strain DSM 44595 16S ribosomal RNA... | NR_104776.1 | 100 | 335 | 0 | 2.30E-177 |
| Feces slurry | 14P 4.3_Bac1 | E | 30 | Serratia liquefaciens strain ATCC 27592 16S ribosoma... | NR_121703.1 | 100 | 411 | 0 | 0 |
| Feces slurry | 14P 4.3_Bac2 | P | 31 | Chryseobacterium rhizosphaerae strain RSB3-1 16S rib... | NR_125812.1 | 99.507 | 406 | 1 | 0 |
| Feces slurry | 14P 4.3_Bac3 | I | 166 | Pseudomonas protegens strain CHA0 16S ribosomal RNA ... | NR_114749.1 | 100 | 411 | 0 | 0 |
| Feces slurry | 14P 4.3_Bac4 | I | 167 | Pseudomonas protegens strain CHA0 16S ribosomal RNA ... | NR_114749.1 | 100 | 411 | 0 | 0 |
| Feces slurry | 14P 5.3_Bac1 | E | 32 | Pseudomonas fluorescens strain NBRC 14160 16S riboso... | NR_113647.1 | 100 | 411 | 0 | 0 |
| Feces slurry | 14P 5.3_Bac2 | E | 33 | Pseudomonas migulae strain NBRC 103157 16S ribosomal... | NR_114223.1 | 99.513 | 411 | 2 | 0 |
| Feces slurry | 14P 6.1_Bac1 | I | 34 | Rhodococcus globerulus strain DSM 43954 16S ribosoma... | NR_118617.1 | 100 | 390 | 0 | 0 |
| Feces slurry | 14P 6.2_Bac1 | G | 168 | Microbacterium maritypicum strain DSM 12512 16S ribo... | NR_114986.1 | 99.744 | 390 | 1 | 0 |
| Feces slurry | 14P 6.2_Bac2 | G | 169 | Microbacterium maritypicum strain DSM 12512 16S ribo... | NR_114986.1 | 99.744 | 390 | 1 | 0 |
| Feces slurry | 14P 6.2_Bac3 | E | 170 | Stenotrophomonas maltophilia strain ATCC 13637 16S r... | NR_112030.1 | 98.054 | 411 | 8 | 0 |
| Feces slurry | 14P 6.3_Bac1 | E | 171 | Stenotrophomonas maltophilia strain ATCC 13637 16S r... | NR_112030.1 | 98.054 | 411 | 8 | 0 |
| Feces slurry | 14P 7.1_Bac1 | Poor growth; not frozen; no edibility | 35 | Paenibacillus tundrae strain A10b 16S ribosomal RNA ... | NR_044525.1 | 100 | 410 | 0 | 0 |
| Feces slurry | 14P 7.1_Bac2 | G | 36 | Paenibacillus pabuli strain NBRC 13638 16S ribosomal... | NR_113627.1 | 100 | 410 | 0 | 0 |
| Feces slurry | 14P 7.1_Bac3 | Poor growth; not frozen; no edibility | 37 | Paenibacillus pabuli strain NBRC 13638 16S ribosomal... | NR_113627.1 | 100 | 410 | 0 | 0 |
| Feces slurry | 14P 7.1_Bac4 | Poor growth; not frozen; no edibility | 38 | Paenibacillus pabuli strain NBRC 13638 16S ribosomal... | NR_113627.1 | 100 | 410 | 0 | 0 |
| Feces slurry | 14P 7.2_Bac1 | P | 40 | Paenibacillus pabuli strain NBRC 13638 16S ribosomal... | NR_113627.1 | 100 | 410 | 0 | 0 |
| Feces slurry | 14P 7.2_Bac2 | Poor growth; not frozen; no edibility | 41 | Paenibacillus endophyticus strain PECAE04 16S riboso... | NR_135705.1 | 99.51 | 408 | 2 | 0 |
| Feces slurry | 14P 8.1_Bac1 | E | 42 | Pseudomonas kuykendallii strain H2 16S ribosomal RNA... | NR_118155.1 | 98.783 | 411 | 5 | 0 |
| Feces slurry | 14P 8.1_Bac2 | E | 172 | Chryseobacterium rhizosphaerae strain RSB3-1 16S rib... | NR_125812.1 | 99.507 | 406 | 1 | 0 |
| Feces slurry | 14P 8.1_Bac3 | E | 44 | Pseudomonas helmanticensis strain OHA11 16S ribosoma... | NR_126220.1 | 99.27 | 411 | 3 | 0 |
| Feces slurry | 14P 8.1_Bac4 | E | 45 | Comamonas kerstersii strain LMG 3475 16S ribosomal R... | NR_025530.1 | 99.27 | 411 | 3 | 0 |
| Feces slurry | 14P 10.2_Bac2 | G | 47b | Paenibacillus pabuli strain NBRC 13638 16S ribosomal... | NR_113627.1 | 100 | 410 | 0 | 0 |
| Feces slurry | 17P 9.1_Bac1 | G | 48 | Rhodococcus agglutinans strain CFH S0262 16S ribosom... | NR_136860.1 | 98.977 | 391 | 3 | 0 |
| Feces slurry | 17P 9.1_Bac2 | Poor growth; not frozen; no edibility | 173 | Paenibacillus pabuli strain NBRC 13638 16S ribosomal... | NR_113627.1 | 100 | 410 | 0 | 0 |
| Feces slurry | 18P 2.1_Bac1 | P | 174 | Microbacterium maritypicum strain DSM 12512 16S ribo... | NR_114986.1 | 99.507 | 406 | 1 | 0 |
| Feces slurry | 18P 2.1_Bac2 | G | 175 | Paenibacillus pabuli strain NBRC 13638 16S ribosomal... | NR_113627.1 | 100 | 410 | 0 | 0 |
| Feces slurry | 18P 2.1_Bac3 | I | 176 | Rhodococcus globerulus strain DSM 43954 16S ribosoma... | NR_118617.1 | 100 | 390 | 0 | 0 |
| Feces slurry | 18P 2.2_Bac1 | E | 49 | Agrobacterium tumefaciens strain IAM 12048 16S ribos... | NR_041396.1 | 100 | 386 | 0 | 0 |
| Feces slurry | 18P 3.1_Bac1 | P | 179 | Chryseobacterium rhizosphaerae strain RSB3-1 16S rib... | NR_125812.1 | 99.507 | 406 | 1 | 0 |
| Feces slurry | 18P 4.2_Bac1 | E | 50 | Escherichia fergusonii strain ATCC 35469 16S ribosom... | NR_074902.1 | 100 | 411 | 0 | 0 |
| Feces slurry | 18P 4.2_Bac2 | P | 51 | Paenibacillus pabuli strain NBRC 13638 16S ribosomal... | NR_113627.1 | 100 | 410 | 0 | 0 |
| Feces slurry | 18P 4.2_Bac3 | I | 52 | Nocardia coeliaca strain DSM 44595 16S ribosomal RNA... | NR_104776.1 | 100 | 390 | 0 | 0 |
| Feces slurry | 18P 5.1_Bac1 | E | 182 | Stenotrophomonas pavanii strain LMG 25348 16S riboso... | NR_118008.1 | 97.81 | 411 | 9 | 0 |
| Feces slurry | 18P 5.1_Bac2 | I | 183 | Nocardia coeliaca strain DSM 44595 16S ribosomal RNA... | NR_104776.1 | 100 | 390 | 0 | 0 |
| Feces slurry | 18P 5.1_Bac3 | I | 184 | Nocardia coeliaca strain DSM 44595 16S ribosomal RNA... | NR_104776.1 | 100 | 390 | 0 | 0 |
| Feces slurry | 18P 6.1_Bac1 | G | 53 | Paenibacillus pabuli strain NBRC 13638 16S ribosomal... | NR_113627.1 | 100 | 410 | 0 | 0 |
| Feces slurry | 18P 6.1_Bac2 | G | 185 | Paenibacillus pabuli strain NBRC 13638 16S ribosomal... | NR_113627.1 | 100 | 410 | 0 | 0 |
| Feces slurry | 18P 6.2_Bac1 | G | 54 | Microbacterium maritypicum strain DSM 12512 16S ribo... | NR_114986.1 | 99.744 | 390 | 1 | 0 |
| Feces slurry | 18P 6.2_Bac2 | I | 55 | Nocardia coeliaca strain DSM 44595 16S ribosomal RNA... | NR_104776.1 | 100 | 385 | 0 | 0 |
| Feces slurry | 18P 6.2_Bac3 | E | 56 | Comamonas testosteroni strain NBRC 14951 16S ribosom... | NR_113709.1 | 99.515 | 412 | 1 | 0 |
| Feces slurry | 18P 6.2_Bac4 | I | 186 | Kaistia terrae strain 5YN7-3 16S ribosomal RNA gene,... | NR_108222.1 | 99.751 | 401 | 0 | 0 |
| Feces slurry | 18P 6.2_Bac5 | E | 187 | Paenibacillus pabuli strain NBRC 13638 16S ribosomal... | NR_113627.1 | 100 | 410 | 0 | 0 |
| Feces slurry | 18P 6.2_Bac6 | G | 188 | Pseudomonas koreensis strain Ps 9-14 16S ribosomal R... | NR_025228.1 | 100 | 411 | 0 | 0 |
| Feces slurry | 18P 8.1_Bac1 | E | 57 | Achromobacter aegrifaciens strain LMG 26852 16S ribo... | NR_117707.1 | 99.757 | 411 | 1 | 0 |
| Feces slurry | 18P 8.2_Bac1 | I | 58 | Pseudomonas protegens strain CHA0 16S ribosomal RNA ... | NR_114749.1 | 100 | 411 | 0 | 0 |
| Feces slurry | 18P 8.2_Bac2 | E | 59 | Buttiauxella warmboldiae strain NSW 326 16S ribosoma... | NR_028893.1 | 99.513 | 411 | 2 | 0 |
| Feces slurry | 18P 10.1_Bac1 | G | 189 | Paenibacillus pabuli strain NBRC 13638 16S ribosomal... | NR_113627.1 | 100 | 410 | 0 | 0 |
| Feces slurry | 18P 10.1_Bac2 | P | 190 | Williamsia marianensis strain DSM 44944 16S ribosoma... | NR_118613.1 | 100 | 390 | 0 | 0 |
| Feces slurry | 19P 8.1_Bac1 | P | 191 | Paenibacillus pabuli strain NBRC 13638 16S ribosomal... | NR_113627.1 | 100 | 410 | 0 | 0 |
| Feces slurry | 19P 9.1_Bac1 | P | 61 | Paenibacillus pabuli strain NBRC 13638 16S ribosomal... | NR_113627.1 | 100 | 410 | 0 | 0 |
| Feces slurry | 19P 10.1_Bac1 | I | 62 | Paenibacillus pabuli strain NBRC 13638 16S ribosomal... | NR_113627.1 | 100 | 410 | 0 | 0 |
| Feces slurry | 19P 10.1_Bac2 | G | 63 | Paenibacillus pabuli strain NBRC 13638 16S ribosomal... | NR_113627.1 | 100 | 410 | 0 | 0 |
| Feces slurry | 20P 2.1_Bac1 | E | 64 | Paenibacillus pabuli strain NBRC 13638 16S ribosomal... | NR_113627.1 | 100 | 410 | 0 | 0 |
| Feces slurry | 20P 2.1_Bac2 | G | 65 | Pandoraea oxalativorans strain TA25 16S ribosomal RN... | NR_112832.1 | 100 | 411 | 0 | 0 |
| Feces slurry | 20P 3.2_Bac1 | P | 67 | Paenibacillus pabuli strain NBRC 13638 16S ribosomal... | NR_113627.1 | 100 | 410 | 0 | 0 |
| Feces slurry | 20P 3.2_Bac2 | E | 68 | Flavobacterium ginsengisoli strain DCY54 16S ribosom... | NR_109024.1 | 100 | 405 | 0 | 0 |
| Feces slurry | 20P 3.2_Bac3 | P | 69 | Paenibacillus pabuli strain NBRC 13638 16S ribosomal... | NR_113627.1 | 100 | 410 | 0 | 0 |
| Feces slurry | 20P 3.2_Bac4 | I | 70 | Pseudomonas vranovensis strain 2B2 16S ribosomal RNA... | NR_043313.1 | 100 | 411 | 0 | 0 |
| Feces slurry | 20P 3.2_Bac5 | I | 71 | Pseudomonas vranovensis strain 2B2 16S ribosomal RNA... | NR_043313.1 | 100 | 411 | 0 | 0 |
| Feces slurry | 20P 3.2_Bac6 | P | 72 | Paenibacillus pabuli strain NBRC 13638 16S ribosomal... | NR_113627.1 | 100 | 410 | 0 | 0 |
| Feces slurry | 20P 6.1_Bac1 | E | 73 | Stenotrophomonas pavanii strain LMG 25348 16S riboso... | NR_118008.1 | 97.81 | 411 | 9 | 0 |
| Feces slurry | 20P 6.1_Bac2 | E | 74 | Brucella papionis strain F8/08-60 16S ribosomal RNA,... | NR_133990.1 | 99.223 | 386 | 3 | 0 |
| Feces slurry | 20P 6.1_Bac3 | I | 75 | Rhodococcus jialingiae strain djl-6-2 16S ribosomal ... | NR_115708.1 | 100 | 322 | 0 | 3.72E-170 |
| Feces slurry | 20P 7.2_Bac1 | G | 76 | Bacillus aryabhattai strain B8W22 16S ribosomal RNA ... | NR_118442.1 | 99.513 | 411 | 2 | 0 |
| Feces slurry | 20P 7.2_Bac2 | G | 77 | Bacillus aryabhattai strain B8W22 16S ribosomal RNA ... | NR_118442.1 | 99.513 | 411 | 2 | 0 |
| Feces slurry | 20P 9.1_Bac1 | E | 78 | Shinella zoogloeoides strain NBRC 102405 16S ribosom... | NR_114067.1 | 100 | 386 | 0 | 0 |
| Feces slurry | 20P 9.1_Bac2 | E | 79 | Agrobacterium rubi strain NBRC 13261 16S ribosomal R... | NR_113608.1 | 100 | 386 | 0 | 0 |
| Feces slurry | 20P 10.1_Bac1 | E | 192 | Paenibacillus pabuli strain NBRC 13638 16S ribosomal... | NR_113627.1 | 100 | 410 | 0 | 0 |
| Feces slurry | 20P 10.2_Bac1 | E | 80 | Sphingobacterium ginsenosidimutans strain THG 07 16S... | NR_108689.1 | 99.012 | 405 | 4 | 0 |
| Feces slurry | 20P 10.2_Bac2 | G | 81 | Rhizobium endophyticum strain CCGE 2052 16S ribosoma... | NR_116477.1 | 99.482 | 386 | 2 | 0 |
| Feces slurry | 20P 10.2_Bac3 | G | 82 | Shinella zoogloeoides strain NBRC 102405 16S ribosom... | NR_114067.1 | 100 | 318 | 0 | 6.14E-168 |
| Feces slurry | 20P 10.2_Bac4 | E | 83 | Variovorax boronicumulans strain NBRC 103145 16S rib... | NR_114214.1 | 99.027 | 411 | 4 | 0 |
| Feces slurry | 20P 10.2_Bac5 | I | 84 | Paenibacillus pabuli strain NBRC 13638 16S ribosomal... | NR_113627.1 | 100 | 410 | 0 | 0 |
| Feces ball | 13B 2.1_Bac1 | E | 141 | Pseudomonas palleroniana strain CFBP 4389 16S riboso... | NR_029050.1 | 100 | 411 | 0 | 0 |
| Feces ball | 13B 3.1_Bac1 | E | 206 | Oxalicibacterium horti strain NBRC 13594 16S ribosom... | NR_113620.1 | 99.027 | 411 | 4 | 0 |
| Feces ball | 13B 3.1_Bac2 | G | 207 | Oxalicibacterium horti strain NBRC 13594 16S ribosom... | NR_113620.1 | 99.027 | 411 | 4 | 0 |
| Feces ball | 13B 3.2_Bac1 | E | 142 | Pseudomonas migulae strain NBRC 103157 16S ribosomal... | NR_114223.1 | 99.513 | 411 | 2 | 0 |
| Feces ball | 13B 5.1_Bac1 | E | 146 | Paenibacillus pabuli strain NBRC 13638 16S ribosomal... | NR_113627.1 | 100 | 410 | 0 | 0 |
| Feces ball | 13B 5.1_Bac2 | Poor growth; not frozen; no edibility | 208 | Paenibacillus pabuli strain NBRC 13638 16S ribosomal... | NR_113627.1 | 100 | 410 | 0 | 0 |
| Shallow soil | 2S 7.1_Bac1 | G | 85 | Paenibacillus pabuli strain NBRC 13638 16S ribosomal... | NR_113627.1 | 100 | 410 | 0 | 0 |
| Shallow soil | 3S 7.1_Bac1 | G | 86 | Paenibacillus pabuli strain NBRC 13638 16S ribosomal... | NR_113627.1 | 100 | 407 | 0 | 0 |
| Shallow soil | 3S 7.1_Bac2 | E | 87 | Pandoraea oxalativorans strain TA25 16S ribosomal RN... | NR_112832.1 | 100 | 411 | 0 | 0 |
| Shallow soil | 4S 1.1_Bac1 | E | 88 | Serratia quinivorans strain 4364 16S ribosomal RNA g... | NR_037112.1 | 100 | 411 | 0 | 0 |
| Shallow soil | 4S 2.1_Bac1 | I | 89 | Paraburkholderia metrosideri strain DNBP6-1 16S ribo... | NR_146373.1 | 99.757 | 412 | 0 | 0 |
| Shallow soil | 4S 2.1_Bac2 | E | 90 | Burkholderia ginsengisoli strain NBRC 100965 16S rib... | NR_113964.1 | 100 | 411 | 0 | 0 |
| Shallow soil | 4S 4.1_Bac1 | E | 91 | Serratia quinivorans strain 4364 16S ribosomal RNA g... | NR_037112.1 | 100 | 411 | 0 | 0 |
| Shallow soil | 4S 4.1_Bac2 | E | 92 | Rahnella inusitata strain DSM 30078 16S ribosomal RN... | NR_146846.1 | 99.757 | 411 | 1 | 0 |
| Shallow soil | 4S 4.1_Bac3 | E | 194 | Serratia quinivorans strain 4364 16S ribosomal RNA g... | NR_037112.1 | 100 | 411 | 0 | 0 |
| Shallow soil | 5S 1.2_Bac1 | I | 93 | Pseudomonas migulae strain NBRC 103157 16S ribosomal... | NR_114223.1 | 99.513 | 411 | 2 | 0 |
| Shallow soil | 5S 2.1_Bac1 | E | 94 | Staphylococcus saprophyticus subsp. bovis strain GTC... | NR_041324.1 | 100 | 411 | 0 | 0 |
| Shallow soil | 5S 7.1_Bac1 | G | 95 | Burkholderia fungorum strain LMG 16225 16S ribosomal... | NR_118060.1 | 99.757 | 411 | 1 | 0 |
| Shallow soil | 5S 10.2_Bac1 | P | 195 | Paenibacillus pabuli strain NBRC 13638 16S ribosomal... | NR_113627.1 | 100 | 336 | 0 | 6.42E-178 |
| Shallow soil | 9S 1.1_Bac1 | I | 96 | Burkholderia phenazinium strain LMG 2247 16S ribosom... | NR_118071.1 | 99.027 | 411 | 4 | 0 |
| Shallow soil | 9S 5.1_Bac1 | I | 97 | Paraburkholderia metrosideri strain DNBP6-1 16S ribo... | NR_146373.1 | 99.757 | 412 | 0 | 0 |
| Shallow soil | 9S 5.1_Bac2 | Poor growth; not frozen; no edibility | 196 | Paenibacillus pabuli strain NBRC 13638 16S ribosomal... | NR_113627.1 | 100 | 336 | 0 | 6.42E-178 |
| Shallow soil | 9S 6.2_Bac1 | I | 98 | Paraburkholderia metrosideri strain DNBP6-1 16S ribo... | NR_146373.1 | 99.757 | 412 | 0 | 0 |
| Shallow soil | 9S 7.1_Bac1 | P | 99 | Burkholderia fungorum strain LMG 16225 16S ribosomal... | NR_118060.1 | 99.757 | 411 | 1 | 0 |
| Shallow soil | 16S 3.1_Bac1 | P | 102 | Bacillus toyonensis strain BCT-7112 16S ribosomal RN... | NR_121761.1 | 100 | 412 | 0 | 0 |
| Shallow soil | 16S 3.1_Bac2 | E | 103 | Paenibacillus pabuli strain NBRC 13638 16S ribosomal... | NR_113627.1 | 100 | 410 | 0 | 0 |
| Shallow soil | 16S 3.1_Bac3 | G | 104 | Paenibacillus pabuli strain NBRC 13638 16S ribosomal... | NR_113627.1 | 100 | 409 | 0 | 0 |
| Shallow soil | 16S 3.1_Bac4 | G | 105 | Paenibacillus pabuli strain NBRC 13638 16S ribosomal... | NR_113627.1 | 100 | 410 | 0 | 0 |
| Shallow soil | 16S 3.1_Bac5 | G | 106 | Burkholderia pseudomultivorans strain LMG 26883 16S ... | NR_117661.1 | 99.027 | 411 | 4 | 0 |
| Shallow soil | 16S 4.1_Bac1 | Poor growth; not frozen; no edibility | 107 | Paenibacillus pabuli strain NBRC 13638 16S ribosomal... | NR_113627.1 | 100 | 410 | 0 | 0 |
| Shallow soil | 16S 7.2_Bac1 | I | 108 | Paraburkholderia metrosideri strain DNBP6-1 16S ribo... | NR_146373.1 | 99.757 | 412 | 0 | 0 |
| Shallow soil | 16S 10.1_Bac1 | P | 109 | Paenibacillus pabuli strain NBRC 13638 16S ribosomal... | NR_113627.1 | 100 | 410 | 0 | 0 |
| Shallow soil | 16S 10.1_Bac2 | P | 110 | Paenibacillus pabuli strain NBRC 13638 16S ribosomal... | NR_113627.1 | 100 | 410 | 0 | 0 |
| Shallow soil | 17S 9.1_Bac1 | E | 111 | Burkholderia ginsengisoli strain NBRC 100965 16S rib... | NR_113964.1 | 100 | 411 | 0 | 0 |
| Shallow soil | 19S 6.1_Bac1 | I | 113 | Nocardia coeliaca strain DSM 44595 16S ribosomal RNA... | NR_104776.1 | 100 | 390 | 0 | 0 |
| Shallow soil | 19S 6.2_Bac1 | P | 197 | Paenibacillus endophyticus strain PECAE04 16S riboso... | NR_135705.1 | 99.512 | 410 | 2 | 0 |
| Shallow soil | 20S 4.1_Bac1 | G | 198 | Paenibacillus pabuli strain NBRC 13638 16S ribosomal... | NR_113627.1 | 100 | 336 | 0 | 6.42E-178 |
| Shallow soil | 20S 6.1_Bac1 | I | 114 | Rhodococcus globerulus strain DSM 43954 16S ribosoma... | NR_118617.1 | 100 | 390 | 0 | 0 |
| Shallow soil | 20S 6.1_Bac2 | G | 115 | Pandoraea oxalativorans strain TA25 16S ribosomal RN... | NR_112832.1 | 100 | 411 | 0 | 0 |
| Shallow soil | 20S 6.2_Bac1 | I | 116 | Pseudomonas fragi strain NBRC 3458 16S ribosomal RNA... | NR_113578.1 | 99.027 | 411 | 4 | 0 |
| Shallow soil | 20S 6.3_Bac1 | P | 118 | Paenibacillus pabuli strain NBRC 13638 16S ribosomal... | NR_113627.1 | 100 | 410 | 0 | 0 |
| Shallow soil | 20S 7.1_Bac1 | G | 199 | Paenibacillus taohuashanense strain gs65 16S ribosom... | NR_118393.1 | 100 | 380 | 0 | 0 |
| Deep soil | 1D 2.1_Bac1 | P | 119 | Paenibacillus pabuli strain NBRC 13638 16S ribosomal... | NR_113627.1 | 100 | 410 | 0 | 0 |
| Deep soil | 1D 2.1_Bac2 | Poor growth; not frozen; no edibility | 120 | Paenibacillus pabuli strain NBRC 13638 16S ribosomal... | NR_113627.1 | 100 | 410 | 0 | 0 |
| Deep soil | 2D 7.1_Bac1 | P | 200 | Paenibacillus pabuli strain NBRC 13638 16S ribosomal... | NR_113627.1 | 100 | 410 | 0 | 0 |
| Deep soil | 2D 7.1_Bac2 | P | 201 | Paenibacillus pabuli strain NBRC 13638 16S ribosomal... | NR_113627.1 | 100 | 410 | 0 | 0 |
| Deep soil | 2D 7.1_Bac3 | G | 202 | Paenibacillus pabuli strain NBRC 13638 16S ribosomal... | NR_113627.1 | 100 | 410 | 0 | 0 |
| Deep soil | 2D 10.1_Bac1 | P | 121 | Paenibacillus pabuli strain NBRC 13638 16S ribosomal... | NR_113627.1 | 100 | 410 | 0 | 0 |
| Deep soil | 2D 10.1_Bac2 | G | 122 | Paenibacillus pabuli strain NBRC 13638 16S ribosomal... | NR_113627.1 | 100 | 404 | 0 | 0 |
| Deep soil | 2D 10.1_Bac3 | P | 123 | Paenibacillus pabuli strain NBRC 13638 16S ribosomal... | NR_113627.1 | 100 | 407 | 0 | 0 |
| Deep soil | 2D 10.1_Bac4 | P | 124 | Paenibacillus pabuli strain NBRC 13638 16S ribosomal... | NR_113627.1 | 100 | 410 | 0 | 0 |
| Deep soil | 3D 8.3_Bac1 | G | 126 | Paenibacillus pabuli strain NBRC 13638 16S ribosomal... | NR_113627.1 | 100 | 407 | 0 | 0 |
| Deep soil | 6D 1.1_Bac1 | G | 127 | Paenibacillus pabuli strain NBRC 13638 16S ribosomal... | NR_113627.1 | 100 | 410 | 0 | 0 |
| Deep soil | 6D 1.1_Bac2 | Poor growth; not frozen; no edibility | 203 | Paenibacillus pabuli strain NBRC 13638 16S ribosomal... | NR_113627.1 | 100 | 410 | 0 | 0 |
| Deep soil | 6D 1.2_Bac1 | G | 128 | Burkholderia fungorum strain LMG 16225 16S ribosomal... | NR_118060.1 | 99.757 | 411 | 1 | 0 |
| Deep soil | 6D 2.1_Bac1 | P | 129 | Paenibacillus pabuli strain NBRC 13638 16S ribosomal... | NR_113627.1 | 100 | 397 | 0 | 0 |
| Deep soil | 6D 7.1_Bac1 | P | 130 | Pseudomonas lini strain DLE411J 16S ribosomal RNA ge... | NR_029042.2 | 99.272 | 412 | 1 | 0 |
| Deep soil | 6D 7.1_Bac2 | E | 131 | Burkholderia pseudomultivorans strain LMG 26883 16S ... | NR_117661.1 | 99.027 | 411 | 4 | 0 |
| Deep soil | 6D 8.1_Bac1 | E | 132 | Burkholderia phenazinium strain ATCC 33666 16S ribos... | NR_112068.1 | 99.513 | 411 | 2 | 0 |
| Deep soil | 6D 8.1_Bac2 | P | 133 | Burkholderia phenazinium strain ATCC 33666 16S ribos... | NR_112068.1 | 99.513 | 411 | 2 | 0 |
| Deep soil | 6D 8.2_Bac1 | G | 134 | Burkholderia sediminicola strain HU2-65W 16S ribosom... | NR_044383.1 | 100 | 411 | 0 | 0 |
| Deep soil | 12D 9.1_Bac1 | G | 136 | Burkholderia phenazinium strain ATCC 33666 16S ribos... | NR_112068.1 | 98.783 | 411 | 5 | 0 |
| Deep soil | 12D 9.1_Bac2 | G | 137 | Burkholderia ginsengisoli strain NBRC 100965 16S rib... | NR_113964.1 | 99.027 | 411 | 4 | 0 |
| Deep soil | 17D 6.2_Bac1 | Poor growth; not frozen; no edibility | 204 | Paenibacillus tundrae strain A10b 16S ribosomal RNA ... | NR_044525.1 | 100 | 336 | 0 | 6.42E-178 |
| Deep soil | 17D 8.1_Bac1 | Poor growth; not frozen; no edibility | 205 | Paenibacillus pabuli strain NBRC 13638 16S ribosomal... | NR_113627.1 | 100 | 336 | 0 | 6.42E-178 |
| Deep soil | 17D 9.2_Bac1 | I | 139 | Bacillus toyonensis strain BCT-7112 16S ribosomal RN... | NR_121761.1 | 100 | 412 | 0 | 0 |
| Deep soil | 17D 10.1_Bac1 | G | 140 | Burkholderia fungorum strain LMG 16225 16S ribosomal... | NR_118060.1 | 99.757 | 411 | 1 | 0 |
